# Synthesis, anti-microbial activity, cytotoxicity of some novel substituted (5-(3-(1*H*-benzo[d]imidazol-2-yl)-4-hydroxybenzyl)benzofuran-2-yl)(phenyl)methanone analogs

**DOI:** 10.1186/s13065-017-0364-3

**Published:** 2018-01-09

**Authors:** Bhookya Shankar, Pochampally Jalapathi, Balabadra Saikrishna, Shaym Perugu, Vijjulatha Manga

**Affiliations:** 1 Department of Chemistry, University College of Science, Osmania University, Hyderabad, Telangana India; 2 Molecular Modelling and Medicinal Chemistry Group, Department of Chemistry, Osmania University, Hyderabad, Telangana India; 3 Biomedical Informatics Centre, National Institute of Nutrition, Hyderabad, Telangana India

**Keywords:** Benzimidazoles, Antimicrobial activity, Cytotoxicity, Docking studies

## Abstract

**Background:**

There is a dire need for the discovery and development of new antimicrobial agents after several experiments for a better resistance of microorganisms towards antimicrobial agents become a serious health problem for a few years in the past. As benzimidazole possess various types of biological activities, it has been synthesized, in the present study, a new series of (5-(3-(1*H*-benzo[d]imidazol-2-yl)-4-hydroxybenzyl)benzofuran-2-yl)(phenyl)methanone analogs by using the condensation and screened for its in vitro antimicrobial activity and cytotoxicity.

**Results:**

The synthesized (5-(3-(1*H*-benzo[d]imidazol-2-yl)-4-hydroxybenzyl) benzofuran-2-yl)(phenyl)methanone analogs were confirmed by IR, ^1^H and ^13^C-NMR, MS spectra and HRMS spectral data. The synthesized compounds were evaluated for their in vitro antimicrobial potential against Gram-positive (*Bacillus subtilis*, *Bacillus megaterium*, *Staph aureus* and *Streptococcus pyogenes*), Gram-negative (*Escherichia coli*, *Proteus vulgaris*, *Proteus mirabilis* and *Enterobacter aerogenes*) bacterial and fungal (*Aspergillus niger*, *Candida albicans*, *Fusarium oxysporum*, *Fusarium solani*) strains by disc diffusion method and the minimum inhibitory concentration (MIC) in which it has been recorded in microgram per milliliter in comparison to the reference drugs, ciprofloxacin (antibacterial) and nystatin (antifungal). Further, the cytotoxicity (IC_50_ value) has also been assessed on human cervical (HeLa), Supt1 cancer cell lines by using MTT assay.

**Conclusions:**

The following screened compounds (**4d**), (**4f**), (**4g**), (**4k**), (**4l**), (**4o**) and (**4u**) were found to be the best active against all the tested bacterial and fungal strains among all the demonstrated compounds of biological study. The MIC determination was also carried out against bacteria and fungi, the compounds (**4f**) and (**4u**) are found to be exhibited excellent potent against bacteria and fungi respectively. The compounds (**4f**) and (**4u**) were shown non-toxic in nature after screened for cytotoxicity against the cancer cell lines of human cervical (HeLa) and Supt1. Additionally, structure and antibacterial activity relationship were also further supported by in silico molecular docking studies of the active compounds against DNA topoisomerase.
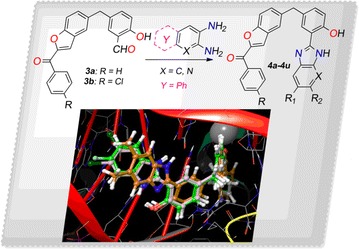

**Electronic supplementary material:**

The online version of this article (10.1186/s13065-017-0364-3) contains supplementary material, which is available to authorized users.

## Background

The innovation and the growth of new antimicrobial and anticancer inhibitory agents are the fundamental goals in medicinal chemistry. According to WHO, number of people affected by cancer will rise from 14 million in 2012 to 22 million within the next 20 years [1]. Most of the cancer cells are defined by unrestrained growth of the cells without differentiation due to the deregulation of essential enzymes and other proteins controlling cell division and proliferation [2, 3]. Clinically, many chemotherapeutic drugs provide a satisfactory response, but they origin a variety of side effects to the patients despite curing the main problem, when they are first exposed to the tumors. Cancer cells have become invulnerable; therefore, there is an urgent need for potential, selective anticancer drugs in contemporary oncology [4]. On the other hand, typhoid, cholera and pneumonia are common worldwide bacterial diseases caused by Gram-negative bacteria. When comparing Gram-positive and Gram-negative bacteria, several species of Gram-negative bacteria are pathogenic. This potential is usually associated with confident components of the walls of Gram-negative cell while exacting the lipopolysaccharide layer [5].

Benzimidazole is a privileged pharmacophore encountered in a number of fundamental cellular components and bioactive molecules. Indeed, a number of important drugs used in different therapeutic areas contain a benzimidazole moiety [6]. Examples are proton pump inhibitor omeprazole, anti-hypertensive drugs candesartan and telmisartan, anthelmintics albendazole and mebendazole, as well as several other kinds of investigational therapeutic agents including antitumor and anticancer. The substituted benzimidazoles have received considerable interest during preceding two decades as they are endowed with a variety of biological activities and have wide range of therapeutic properties [7]. Benzimidazoles are one of the most proficient heterocyclic moieties, which have active sites in treating various diseases [8]. Frequent reports were published on benzimidazole fragment and its analogues competent to exhibit anticancer as well as antimicrobial activities [9, 10]. The basic moiety of telmisartan (reported as cytotoxicity agent in prostate cancer cell line) is also bis-benzimidazole scaffold. [11]. Literature survey revealed that of the compounds attitude benzimidazole moites reported to possess a number of attractive biological activities such as anti tubercular, anticancer, antihelmintic, anti allergic [12], antihistaminic [13], antifungal [14–16] and anti-inflammatory [17]. Recently, Thomas et al. reported that some novel 2-phenyl benzimidazole were shown cell based assays for cytotoxicity and antiviral activity against the panel of RNA and DNA virus molecules which have been found to be more potent [18]. Benzimidazole related molecules are exhibited to antimicrobial agents in search of the new chemical entities, as there is another approach in which the combination of two or more heterocyclic pharmacophore in the single entity results in more potent activity with different modes of action [19, 20].

**Fig. 1:**
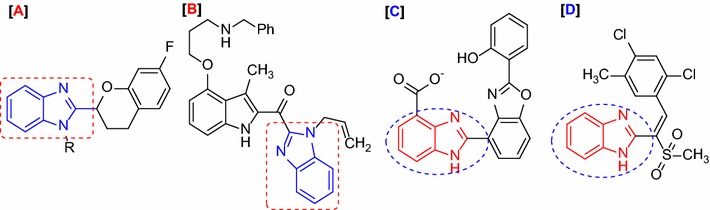
Bioactive benzimidazole scaffolds. **a**, **b** benzimidazole with antimicrobial avtivity and **c**, **d** benzimidazole with anticancer activity

In this present study some novel benzofuran bearing benzimidazole derivatives have been synthesized and their antimicrobial and cytotoxic activities have been established. It was considered worthwhile that to synthesize convinced new chemical entities include two active pharmacophore such as benzimidazoles and benzofuran nucleus single molecular frame work and to get them evaluated for their antimicrobial activity. To the preeminent of our knowledge, this is the best report on the synthesis of benzimidazoles derivatives bearing benzofuran ring. Among the several chemical classes that boast the potential to display the antimicrobial activity [21], cancer activity [22] structures of few benzimidazole possessing significant in vitro antimicrobial (A, B) and cytotoxicity activity (C, D) are presented in Fig. 1.

## Results and discussion

### Chemistry

**Scheme 1:**
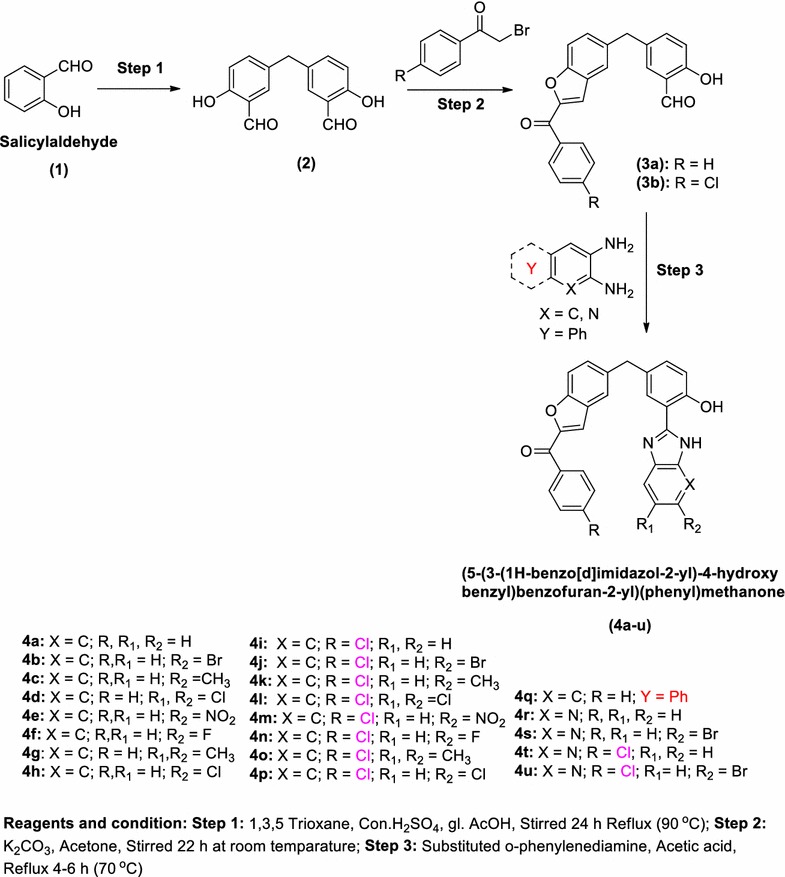
Synthesis of target benzofuran base benzimidazole derivatives **4a**–**u**

For the synthesis of target compounds, 5,5′-methylenebis(2-hydroxybenzaldehyde) (**2**) [23] was prepared in good yield by electrophilic substitution reaction of salicylaldehyde (**1**) with 1,3,5-trioxane(formaldehyde trimer) in glacial acetic acid in the presence of a catalytic amount of concentrated sulfuric acid. Aldehyde (**2**) was subjected to condense cyclisation with substituted phenacyl bromide in the presence of potassium carbonate at the room temperature to obtain the corresponding 5-[(2-benzoylbenzofuran-5-yl)methyl]-2-hydroxybenzaldehyde (**3a**) [24, 25] (Scheme 1). The structures of compound (**3a**) were confirmed by their spectroscopic data (^1^H NMR, ^13^C NMR, IR, MS, and HRMS) which were provided in the experimental part. The ^1^H NMR spectrum of compound (**3a**) showed two singlet signals at 9.85 and 10.98 ppm corresponding to aldehyde and hydroxyl groups, respectively. A singlet signal was suitable to the bridged methylene protons at δ 3.98 ppm, in addition to down field singlet signal due to benzofuran proton and aromatic protons in the region 8.03–6.95 ppm. The conformation regards the structure of the compound (**3a**) is executed by the ^13^C NMR data varying between δ 191.3 and δ 42.09 ppm. The carbon atoms of the two carbonyl groups present at aldehyde and keto appeared more downfield at 191.3 and 182.1 ppm. The two carbon of the –C–O–C– linkage in benzofuran nucleus exhibited the absorption peaks at δ 159.2 and δ 151.4 ppm, respectively. The carbon atoms aromatic ring was observed to exhibit between absorption peaks at δ 154.0–112.1 ppm. The presence of the bridged methylene group between the two aromatic rings was observed to exhibit an absorption peak at δ 42.0 ppm. The IR spectra of the products showed the absorption bands of C=C_str_, HC=O_str_ and –OH_str_ in the region 1650, 1710 and 3550 cm^−1^ respectively. Further confirmation of HRMS spectra showed the obtained peak at m/z = 357.11204 ([M+H]^+^); resultant to a molecular formula C_23_H_17_O_4_.

The synthesis of various compounds (**4a**–**u**) were carried out by condensation of the 5-((2-benzoylbenzofuran-5-yl)methyl)-2-hydroxybenzaldehyde (**3a**) with various substituted ortho phenylenediamine in the presence of glacial acetic acid under conventional reflux temperature in good yield (Scheme 1). The structures of all the synthesized compounds (**4a**–**u**) were thoroughly analyzed by using ^1^H-NMR, ^13^C-NMR, IR, ESI–MS and HRMS analytical techniques. The assigned structures of compound (**4a**) are based on the detailed spectroscopic analysis. The ^1^H NMR spectrum of compound (**4a**) showed singlets of the hydroxyl proton at δ 12.23 and signal of –NH of imidazole ring appeared as singlet at δ 13.18, which was further corroborated through a sharp band at 3420 cm^−1^ in its IR spectrum. ^13^C NMR spectrum of compound (**4a**) showed resonance at δ 182.3 ppm attributed to carbonyl group of benzofuran ring, which was further confirmed by IR spectrum through band at 1740 cm^−1^. The mass spectrum of compound (**4a**) showed peak at m/z 445.08 (M+H)^+^. Further confirmation HRMS spectra showed peak obtained at m/z = found 445.180745 ([M+H]^+^); consequent to a molecular formula C_29_H_21_N_2_O_3_.

In the proton NMR spectrum of (5-(3-(5-fluoro-1*H*-benzo[d]imidazol-2-yl)-4-hydroxybenzyl)benzofuran-2-yl)(phenyl)methanone (**4f**), the signals at δ 13.21 and 12.52 ppm value can be assigned to NH of benzimidazole and OH proton of phenolic moiety, respectively. They multiple at δ 8.02–6.89 ppm in the proton NMR spectrum of compound (**4f**) accounts for three aromatic protons of benzimidazole and phenyl moiety out of the twelve protons. The methylene protons singlet peak assigned at δ 4.01 ppm. In the ^13^C NMR spectrum of compound (**4f**), the chemical signal δ 182.1 and 159.1 ppm were assigned to the carbonyl and hydroxyl aromatic ring respectively. The chemical signals at δ 42.1 were attributed to the bridged methylene carbon. The mass spectrum characterization of the compound (**4f**) exhibits an ion peak at 463.1 m/z which can be designated as the M+1 ion peak and HRMS spectra showed the obtained peak at m/z = 463.14142 ([M+H]^+^), corresponding to a molecular formula C_29_H_20_FN_2_O_3_.

The IR spectrum characterization of compound (5-(3-(5,6-dimethyl-1*H*-benzo[d]imidazol-2-yl)-4-hydroxybenzyl)benzofuran-2-yl)(phenyl) methanone (**4g**) indicated by a shift of absorption band from 3413 cm^−1^, corresponding to the NH group stretching. This can be correlated to the ring stretching of benzimidazole moiety. In the proton NMR spectrum of compound (**4g**), the NH proton has resonated as a singlet at δ 12.95 ppm, OH proton showed as a singlet at δ 9.81 and the multiplet at δ 8.01–6.95 ppm value can be attributed to the aromatic protons of compound (**4g**). The singlet at δ 4.10 and 2.31 ppm was assigned to the bridged methylene protons and two methyl protons respectively. In the ^13^C NMR spectrum of compound (**4g**), the chemical signal δ 183.0 and 151.5 ppm were assigned to the carbonyl and hydroxyl attached carbon respectively. The chemical signals at δ 156.4–112.1 ppm were attributed to the aromatic ring carbons and chemical signals at δ 45.2 and 21.0 ppm bridged methylene protons and two methyl protons respectively. The mass spectrum characterization of the compound (**4g**) exhibits an ion peak at 473.2 m/z which can be designated as the M+1 ion peak and HRMS spectra showed the obtained peak at m/z = 473.18597 ([M+H]^+^) and corresponding to a molecular formula C_31_H_25_N_2_O_3_.

In the proton NMR spectrum of (5-(3-(5-bromo-3*H*-imidazo[4,5-b]pyridin-2-yl)-4-hydroxybenzyl)benzofuran-2-yl)(4-chlorophenyl)methanone (**4u**), the singlet at δ 13.29 and 12.35 ppm was assigned to the NH of the imidazo pyridine and hydroxyl group proton of the phenolic moiety. The thirteen aromatic protons of the compound (**4u**) have resonated at δ 8.20–7.05 ppm as multiplet. The mass spectrum characterization of the compound (**4u**) exhibits an ion peak at 558.9 m/z which can be designated as the M+1 ion peak and HRMS spectra showed the obtained peak at m/z = 557.02621 ([M+H]^+^) consequent to a molecular formula C_29_H_19_O_3_N_2_BrCl.

### In vitro antibacterial activity

The in vitro antibacterial studies of various compounds (**4a**–**u**) were assayed with concentrations (900 μg/mL) against Gram-positive and Gram-negative bacteria by disc diffusion assay [26]. The Gram-positive strains used were *Bacillus subtilis*, *Bacillus megaterium*, *Staph aureus*, *Streptococcus pyogenes* and Gram-negative bacteria were *Escherichia coli*, *Proteus vulgaris*, *Proteus mirabilis* and *Enterobacter aerogenes.* The results were recorded for each weathered compound diameter of inhibition zones of microbial growth around the disks (in mm). The values of zone of inhibition for bacterial are revealed in Table 1. The minimum inhibitory concentration (MIC, μg/mL) is the lowest concentration of a chemical that prevents visible growth of bacterium in microgram per milliliter of active compounds (Table 1) was determined by the microorganism’s susceptibility tests in nutrient and potato dextrose broths were used for the determination of MIC [27]. The results revealed that seven organic compounds displayed better inhibitory effects on the growth of the tested bacterial strains. It was interesting to note that the compounds with substituent on the 3rd position of imidazole ring displayed outstanding antibacterial activity and almost equal to that of standard. However, these trends were transformed closely to antifungal activity.

**Table 1:** Antibacterial screening result of synthesized compounds

Compound code	Zone of inhibition^a^ (mm) and MIC^b^ (μg/mL) of selected compounds							
	Gram-positive				Gram-negative			
	*S. aureus*	*S. pyogenes*	*B. subtilis*	*B. megaterium*	*E. coli*	*P. vulgaris*	*P. mirabilis*	*E. aerogenes*
**4a**	NA	92 (> 100)	121 (> 100)	106 (> 100)	43 (> 100)	31 (> 100)	42 (> 100)	67 (> 100)
**4b**	120 (> 100)	137 (> 100)	36 (> 100)	97 (> 100)	31 (> 100)	96 (> 100)	85 (> 100)	104 (> 100)
**4c**	149 (> 100)	NA	121 (> 100)	103 (> 100)	65 (> 100)	28 (> 100)	61 (> 100)	145 (> 100)
**4d**	102 (75)	92 (25)	98 (50)	125 (25)	114 (25)	75 (25)	197 (25)	118 (25)
**4e**	83 (> 100)	98 (> 100)	NA	92 (> 100)	37 (> 100)	76 (> 100)	93 (> 100)	104 (> 100)
**4f**	210 (50)	167 (50)	181 (50)	121 (25)	67 (25)	141 (75)	193 (25)	124 (25)
**4g**	128 (25)	55 (150)	73 (125)	131 (25)	10 (150)	85 (75)	73 (75)	124 (25)
**4h**	129 (> 100)	44 (> 100)	110 (> 100)	123 (> 100)	90 (> 100)	78 (> 100)	75 (> 100)	124 (> 100)
**4i**	135 (> 100)	52 (> 100)	63 (> 100)	113 (> 100)	58 (> 100)	95 (> 100)	50 (> 100)	57 (> 100)
**4j**	201 (> 100)	114 (> 100)	151 (> 100)	69 (> 100)	51 (> 100)	51 (> 100)	125 (> 100)	98 (> 100)
**4k**	NA	38 (125)	25 (125)	147 (75)	128 (75)	88 (100)	201 (125)	129 (75)
**4l**	195 (50)	92 (75)	56 (150)	79 (75)	140 (100)	28 (50)	25 (50)	52 (25)
**4m**	09 (> 100)	NA	37 (> 100)	88 (> 100)	47 (> 100)	37 (> 100)	37 (> 100)	117 (> 100)
**4n**	57 (> 100)	87 (> 100)	15 (> 100)	60 (> 100)	28 (> 100)	47 (> 100)	127 (> 100)	29 (> 100)
**4o**	128 (50)	116 (75)	NA	120 (125)	58 (125)	9 (125)	156 (75)	73 (25)
**4p**	28 (> 100)	72 (> 100)	53 (> 100)	47 (> 100)	37 (> 100)	12 (> 100)	60 (> 100)	41 (> 100)
**4q**	40 (> 100)	52 (> 100)	37 (> 100)	25 (> 100)	28 (> 100)	57 (> 100)	24 (> 100)	62 (> 100)
**4r**	50 (> 100)	63 (> 100)	24 (> 100)	28 (> 100)	13 (> 100)	43 (> 100)	58 (> 100)	73 (> 100)
**4** **s**	61 (> 100)	39 (> 100)	129 (> 100)	49 (> 100)	65 (> 100)	38 (> 100)	49 (> 100)	109 (> 100)
**4t**	67 (> 100)	38 (> 100)	28 (> 100)	19 (> 100)	25 (> 100)	18 (> 100)	31 (> 100)	36 (> 100)
**4u**	122 (50)	130 (50)	164 (100)	109 (125)	65 (120)	125 (175)	201 (25)	77 (75)
Ciprofloxacin	200 (25)	164 (25)	162 (25)	128 (25)	61 (25)	160 (25)	203 (25)	88 (25)

NA, not active

^a^Zone of inhibition was calculated for stock solution (100 μg/mL)

^b^Minimal inhibitory concentration (MIC) values of the particular compounds are given in brackets

Almost all the synthesized compounds (**4a**–**u**) were found to be active against all investigated pathogenic bacterial strains. As shown in Table 1, it is cleared that the compounds (**4d**), (**4f**), (**4g**), (**4k**), (**4l**), (**4o**) and (**4u**) have superior significant antibacterial potency to the reference drug. In case of *Staphylococcus aureus*, *B. megaterium*, *E. coli*, *E. aerogens*, *P. mirabilis*, compounds (**4d**) and (**4f**) exhibits the highest antibacterial potential with MIC 25–75 μg/mL, it may be attributed that the presence of more electronegative fluoro and dichloro substituent on phenyl ring. The compound (**4u**) displayed significant inhibitory potential with MIC 25 μg/mL against *P. mirabilis*, *P. vulgarise* and threshold activity against *B. megaterium*, and *E. coli* with MIC 120–175 μg/mL, it may have a basic moiety of the bromo pyridine group. In case of *E. aerogens* compounds with methyl (**4c**), dichloro (**4d**) and nitro (**4e**) displayed moderate to be good antibacterial activity with MIC 25 μg/mL. However, the compounds of (**4a**), (**4k**); (**4c**), (**4m**); (**4e**), (**4o**) and (**4g**), (**4r**) were not showed any activity against *S. aureus*, *streptococcus pyogenes*, *and B. subtilis*, respectively; it may be rational owing to the presence of low polar substituents.

### Antifungal activity

The compounds (**4d**), (**4f**), (**4g**), (**4k**), (**4l**), (**4o**) and (**4u**) were tested against four reference fungal strains such as *Aspergillus niger*, *Candida albicans*, *Fusarium oxysporum*, *Fusarium solani* by disc diffusion method. Minimum inhibitory concentration (MIC) in microgram per milliliter of compounds exhibiting activity (Table 2) was determined by the microorganism’s susceptibility tests in nutrient and potato dextrose broths were used for the determination of MIC. The evaluated seven compounds were found to exert a prominent antifungal activity against pathogenic fungal strains. The compounds (**4l**) and (**4u**) exhibited a significant inhibitory activity against *A. niger* and *F. oxysporum* with MIC 25–50 μg/mL, whereas, the compounds with floro (**4f**) and dichloro (**4d**) also exhibited maximum activity with MIC > 25. In case of all fungal strains, compound (**4o**) revealed moderate to good activity with MIC 25–100 μg/mL. The compounds (**4g**) and (**4k**) exhibit less potent inhibitory potential against *A. niger* with an absence of MIC and all the results are given in Table 2. From the obtained in vitro antimicrobial results, it was observed that substitution of electron withdrawing groups at 3rd position of benzimidazole ring leads to increase in both antifungal and antibacterial activity.

**Table 2:** Antifungal screening result of synthesized compounds

Compound code	Zone of inhibition^a^ in (mm) and MIC^b^ (μg/mL)			
	*Aspergillus niger*	*Candida albicans*	*Fusarium oxysporum*	*Fusarium solani*
**4d**	15 (175)	20 (200)	8 (NA)	12 (200)
**4f**	22 (150)	9 (150)	18 (175)	20 (NA)
**4g**	8 (75)	10 (25)	12 (100)	18 (100)
**4k**	9 (NA)	17 (125)	15 (75)	16 (100)
**4l**	22 (NA)	20 (NA)	29 (50)	17 (75)
**4o**	19 (50)	18 (100)	20 (50)	18 (25)
**4u**	26 (25)	19 (25)	20 (25)	18 (50)
Nystatin	23 (25)	27 (25)	28 (25)	28 (25)

NA, not active

^a^Zone of inhibition was calculated for stock solution

^b^Minimal inhibitory concentration (MIC) values of the particular compounds are given in brackets

### In vitro cytotoxicity

MTT assays determine the ability of viable cells to convert a soluble yellow tetrazolium salt (MTT: 3-(4,5-dimethylthiazol-2-yl)-2,5-diphenyl tetrazolium bromide) into insoluble purple formazan crystals by the mitochondrial dehydrogenase enzymes. Cells were exposed to 0.5 mg/mL of MTT for 3 h at 37 °C in an appropriate complete medium. The medium and the MTT were removed after solubilisation in dimethyl sulfoxide (DMSO) and the amount of insoluble formazan crystals was evaluated by measuring the optical density at 550 nm. Each condition was performed in triplicate. Each measurement was corrected from the optical density of MTT alone and expressed relative to the non-treated conditions. Determination of the inhibiting concentration of 50% cell viability and IC_50_ was performed. In short, the fraction of cell affected (Fa) and the fraction of cell unaffected (Fu) relative to one were determined from the viability assay. The log of (Fa/Fu) was plotted against the log of concentration for each compound. Log of IC_50_ was determined at the y-intercept. Standard error was evaluated through the 95% confidence interval.

**Fig. 2:**
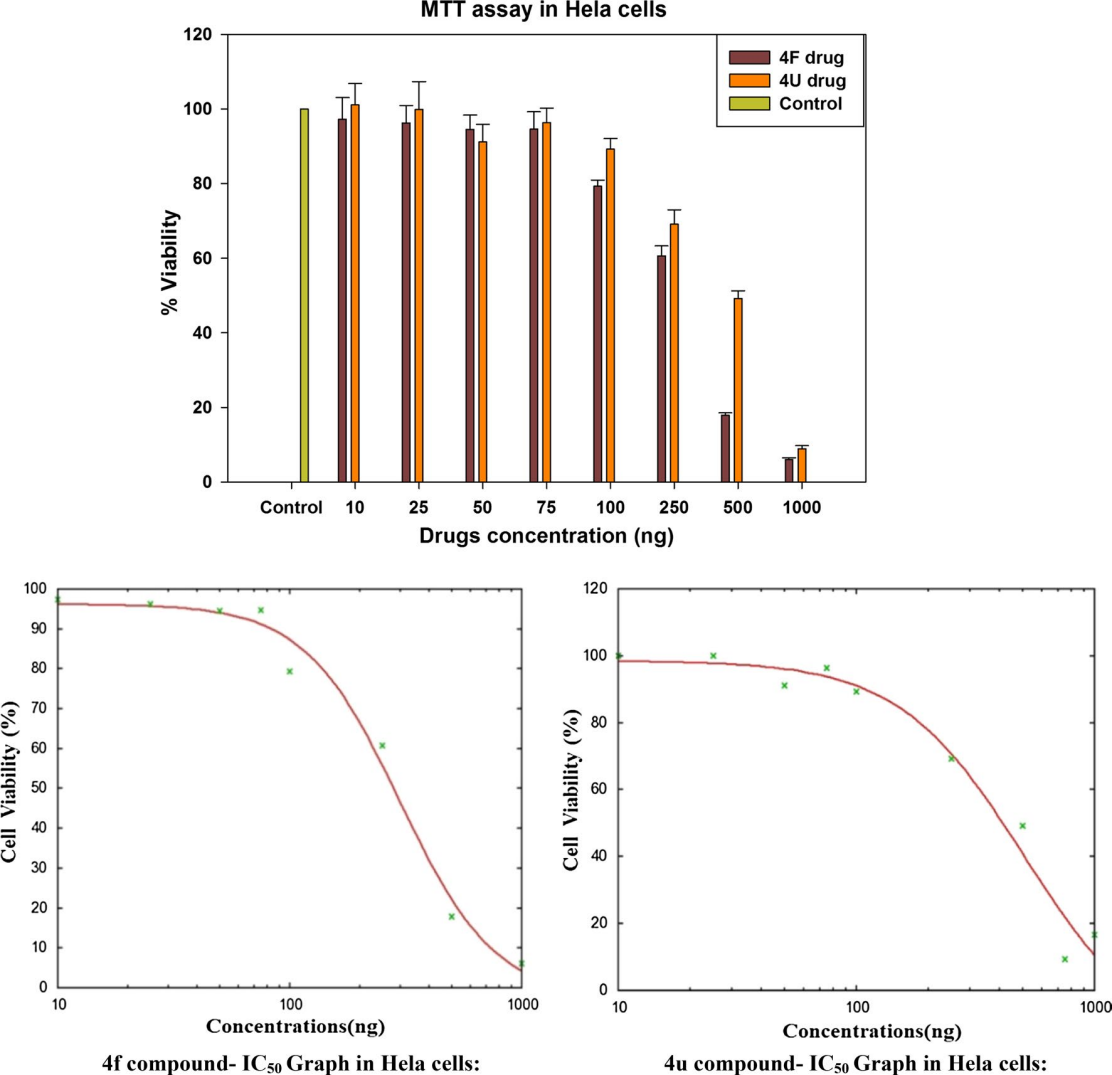
Cell viability assay: hela cells were exposed to increasing concentrations (10, 25, 50, 75, 100, 250, 500 and 1000 ng) of (**4f**) and (**4u**) drugs for 24 h, after which cell viability was determined by MTT assay and their IC_50_ values

**Fig. 3:**
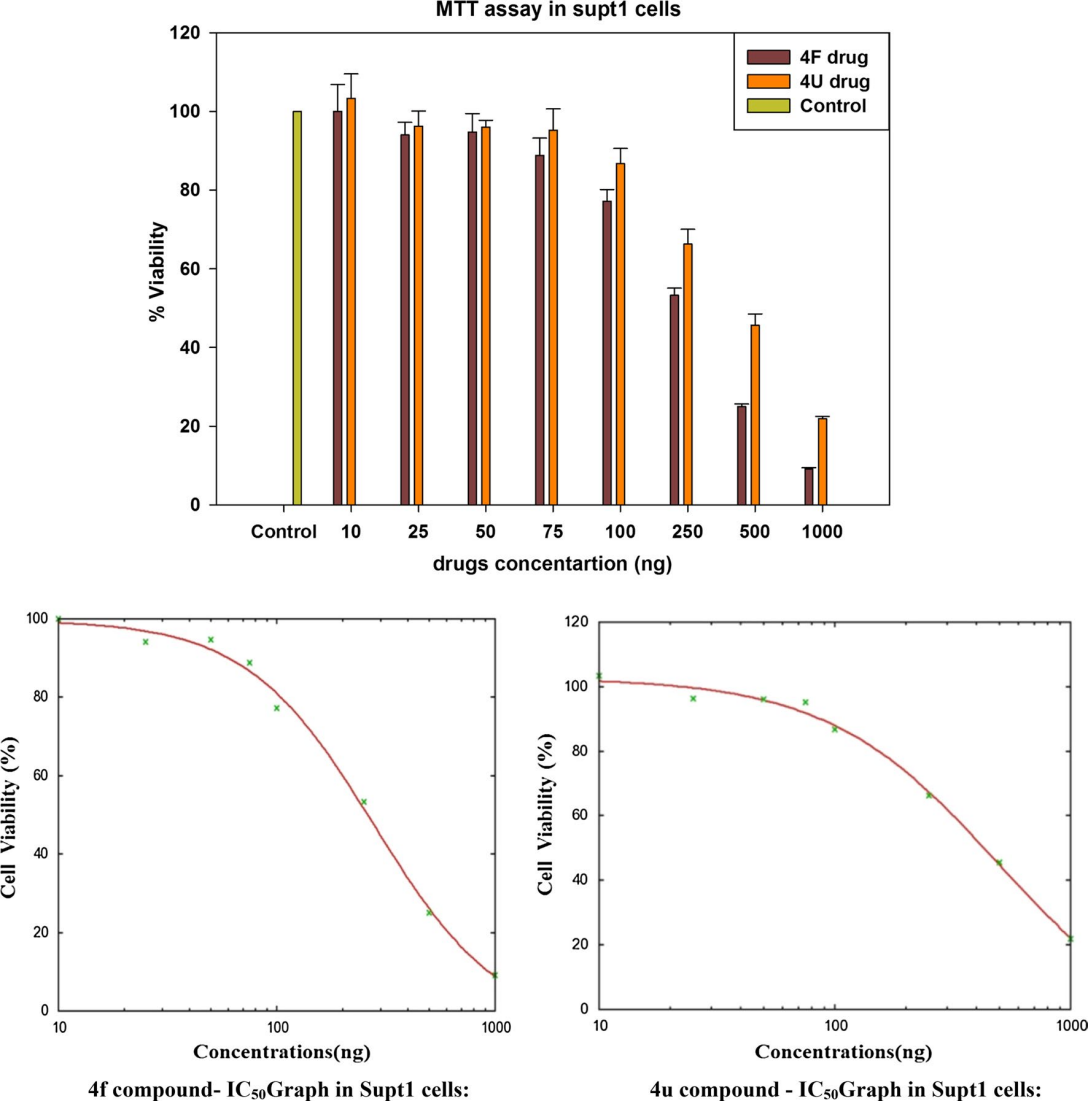
Cell viability assay: Supt1 cells were exposed to increasing concentrations (10, 25, 50, 75, 100, 250, 500 and 1000 ng) of (**4f**) and (**4u**) drugs for 24 h, after which cell viability was determined by MTT assay and their IC_50_ values

The appreciable results were obtained from the previous biological studies, specifically, anti-microbial activity for the derivatives of more microbial active compounds, which encouraged in the present research work to test their cytotoxicity against a human cancer cells (HeLa), Supt1 cell lines. In all the above cases, the activity of the compound (**4f**) and (**4u**) was found to be significantly similar than the controller of the DMSO solvent. To determine the relative cytotoxicity of compounds in the Hela, Supt1 cells were incubated with increasing concentrations (10, 25, 50, 75, 100, 250, 500 and 1000 ng) of the drugs and the survival of the cell was estimated by MTT assay and their IC_50_ were shown in Figs. 2 and 3, respectively. Both the compounds were found non-toxic at very low concentrations (10, 25, 50 and 75 ng), however, the concentration of drugs in about 500 ng was extremely cytotoxic (> 80%). The IC_50_ values at 299.009 and 505.618 ng concentrations in Hela cell lines were shown by the compounds (**4f**) and (**4u**) respectively. The IC_50_ value of compound (**4f**) is 278.73 ng and (**4u**) is 499.903 ng in Supt1 cells. Although the anticancer activity of (**4f**) and (**4u**) is not comparable as standard drug activity, the functional group modifications at the substituted benzimidazole and pyrido imidazole can guide to upgrade the drug leads for further investigations from this laboratory.

Formula for the cell viability of MTT assay

$${\text{Cell viability }} = {{\left( {\text{Test OD}} \right)} \mathord{\left/ {\vphantom {{\left( {\text{Test OD}} \right)} {\left( {\text{Control OD}} \right)}}} \right. \kern-0pt} {\left( {\text{Control OD}} \right)}}\, \times \,100.$$

### Molecular docking

**Fig. 4:**
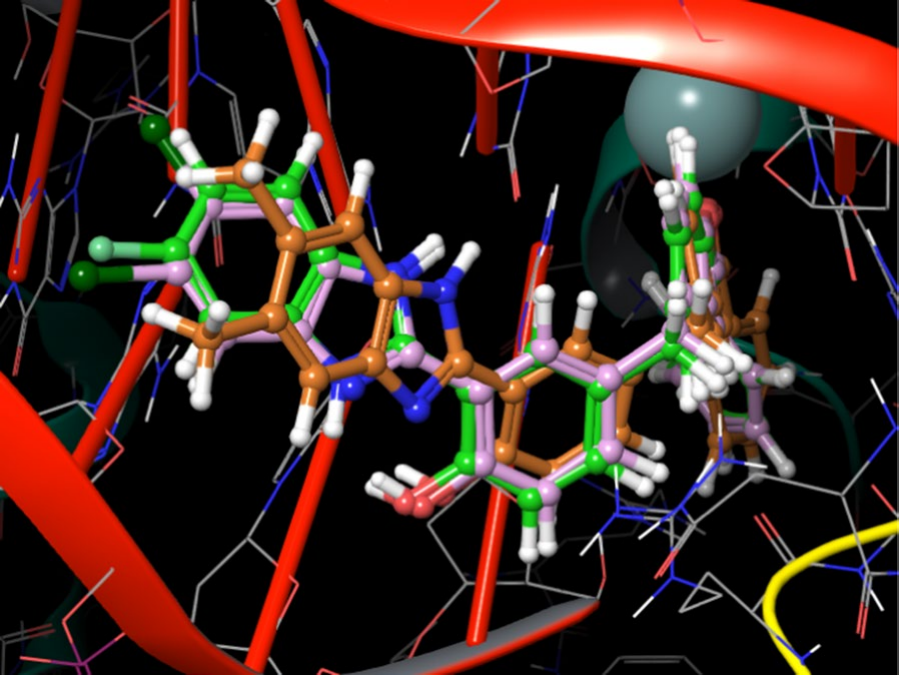
All benzimidazole rings are situated at similar position in the active site pocket of 2XCT

**Fig. 5:**
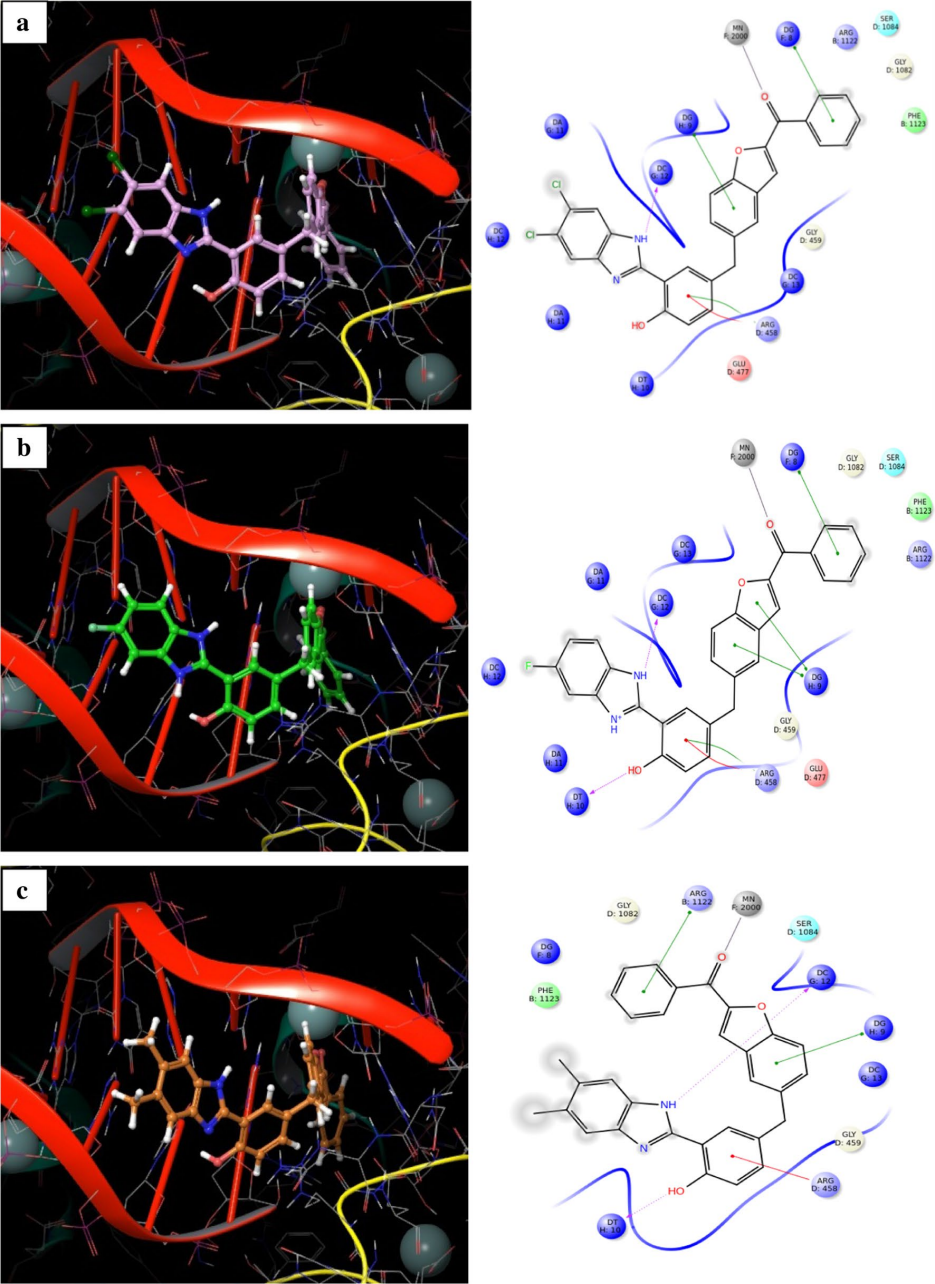
Docking pose and ligand interaction diagram of compounds (**4d**), (**4f**) and (**4g**) (pulm in color **a**, green in color **b** and orange in color **c**) in the active site of 2XCT

The crystal structure of DNA type IIA topoisomerase (pdb id: 2XCT) [28] was retrieved from the Protein Data Bank to understand the interaction between new series of benzimidazole derivatives and type II topoisomerase. The devised software, GLIDE 5.6 [29] was used for molecular docking studies. Protein was prepared by applying default parameters of wizard Maestro 9.0; a grid was generated around the active site by selecting the co-crystalized ligand. Receptor van der Waals scaling for non-polar atoms was kept at 0.9 [30]. The molecules were built by using Maestro build panel and prepared by the application of Lig Prep. Low energy confirmation of the ligands were selected and docked into the grid generated for the protein using the docking mode of extra precision (XP) [31]. Dock pose of each ligand was analyzed for interactions with the receptor. The best and similar interactions with the protein active site were shown from those active molecules, (**4d**), (**4f**) and (**4g**). The molecules were deeply embedded into the hydrophobic active site pocket and they were occupied the similar position as represented in Fig. 4. The docking pose and ligand interaction diagrams of (**4d**), (**4f**) and (**4g**) (pulm in color, green in color and orange in color) are shown in Fig. 5.

To get more insights into the structural basis for its activity, all synthesized compounds were docked into DNA type IIA topoisomerase (2XCT) and the most active compounds (**4d**), (**4f**) and (**4g**) were analyzed in more detail. Docking studies of these compounds showed similar interactions (hydrogen bonds with DC 12 G; DT 10 H, π–π stacking interactions with DG F 8, DG H 9, π–cation interactions with Arg D 458 and metal co-ordination bond with Mn) with the target.

Docking analysis of the best active compounds in the active site of 2XCT revealed that the hydrogen bond, π–π stacking, π–cation and metal co-ordination interactions are important for the activity of these compounds. The obtained result from docking study was well in agreement with experimental results: compound (**4d**) (pulm in color, Fig. 5a) showed only one hydrogen bond with DC G 12 (bond length of 1.893 Å), one π–cation interaction with ARG D 458, two π–π stacking interactions with DG F 8, DG H 9 and metal co-ordination interaction with Mn; whereas compound (**4f**) (green in colour, Fig. 5b) showed two hydrogen bond interactions with DC G 12 (bond length of 1.863 Å), DT H 10 (bond length of 1.916 Å), one π–cation interaction with ARG D 458, three π–π stacking interactions with DG F 8, DG H 9 and metal co-ordination interaction with Mn; compound (**4g**) (orange in color, Fig. 5c) showed two hydrogen bond interactions with DC G 12 (bond length of 1.944 Å), DT H 10 (bond length of 1.905 Å), one π–cation interaction with ARG D 458, two π–π stacking interactions with DG H 9, ARG B 1122 and metal co-ordination interaction with Mn.

From the above results, compound **4d** with one hydrogen bond showed less activity, whereas compounds **4f**, **4g** with two hydrogen bonds showed more activity. These docking results clearly coincided with that of experimental antibacterial activity. Hence, more number of hydrogen bonding interactions with target type II topoisomerase, will affects on more activity. Docking score, glide energy and emodel energies are tabulated in Table 3. Predicted ADME (drug-likeness) properties of all synthesized compounds are provided in Additional file 1.

**Table 3:** Docking score, glide energies and emodel energies of synthesized molecules

Compounds	Docking score (XP)	Glide energy	Glide emodel
**4a**	− 7.35	− 59.84	− 80.02
**4b**	− 7.28	− 56.66	− 83.06
**4c**	− 7.26	− 48.51	− 80.93
**4d**	− *7.37*	− *57.01*	− *81.29*
**4e**	− 7.9	− 62.78	− 92.94
**4f**	− *8.19*	− *60.83*	− *88*
**4g**	− *7.81*	− *66.62*	− *85.31*
**4h**	− 7.03	− 57.46	− 80.99
**4i**	− 7.77	− 61.28	− 85.58
**4j**	− 7.77	− 57.26	− 87.24
**4k**	− 7.05	− 55.46	− 78.16
**4l**	− 7.52	− 57.63	− 88.4
**4m**	− 6.01	− 64.76	− 92.76
**4n**	− 6.6	− 61.97	− 87.16
**4o**	− 6.6	− 56.48	− 84.56
**4p**	− 7.66	− 50.89	− 77.65
**4q**	− 6.19	− 59.74	− 81.1
**4r**	− 6.61	− 59.19	− 84.84
**4s**	− 6.91	− 61.1	− 78.29
**4t**	− 7.28	− 61.13	− 85.76
**4u**	− 7.33	− 57.56	− 84.96

Italic values are the best active compounds (4d, 4f & 4g) with binding energy values like docking score, glide energies and emodel energies

## Experimental

### Chemistry

Melting points are uncorrected and were find out in open capillary tubes in sulphuric acid bath. TLC was carrying out on silica gel-G, and spotting was done using UV light. IR spectra were recorded using Perkin-Elmer 1000 instrument in KBr phase, The ^1^H NMR spectra were record on a Varian as 400 MHz instrument in DMSO, chemical shifts are given in δ ppm relative to TMS, and coupling constants (*J*) are expressed in hertz (Hz). Combinations of the following abbreviations are used to describe NMR spectra: s-singlet; d-doublet; t-triplet; m-multiplet. ^13^C NMR spectra were recorded with a Bruker Advance 400 (100 MHz) spectrometer. Mass spectra on Agilent LC–MS instrument giving only (M^+^+H) values.

#### 5-((2-Benzoylbenzofuran-5-yl)methyl)-2-hydroxybenzaldehyde (**3a**)

The mixture of **3g** compound **2** (0.015 mmol), 1.51 g phenacyl bromide (0.007 mmol), and 3.10 g K_2_CO_3_ (0.022 mmol, 1.5 eq) was stirred in acetone (15 cm^3^) at room temperature for 12 h [24]. The completion of the reaction was monitored by TLC; the product was washed with water (15–25 cm^3^) and extracted from ethyl acetate. The pure compound **3** was separated through column chromatography using petroleum ether/ethyl acetate (70:30, v/v) as white solid (2.7 g, 90%). m.p.: 120–125 °C; ^1^H NMR (400 MHz, CDCl_3_): *δ* = 10.95 (s, 1H, Ar–CHO), 9.85 (s, 1H, OH), 8.03 (d, 2H, *J* = 7.93 Hz, Ar–H), 7.66–7.46 (m, 4H, Ar–H), 7.40–7.30 (m, 5H, Ar–H), 6.95 (d, 2H, *J* = 8.39 Hz, Ar–H), 3.98 (s, 2H, –CH_2_–) ppm; ^13^C NMR (100 MHz, CDCl_3_): *δ* = 191.3, 182.1, 159.2, 154.0, 151.4, 137.9, 137.3, 136.8, 135.3, 132.3, 132.1, 130.9, 129.8 (2C), 128.7 (2C), 126.9, 123.0, 122.0, 117.4, 117.1, 112.1, 42.0 ppm; IR (KBr): $\bar{\nu }$ = 3550, 2800, 1650, 1579, 1720 cm^−1^; MS (ESI+): *m/z* = 357.1 ([M+H]^+^); and HRMS *m/z* calcd for C_23_H_17_O_4_ ([M+H]^+^) 357.11214, found 357.11204.

#### 5-((2-(4-Chlorobenzoyl)benzofuran-5-yl)methyl)-2-hydroxybenzaldehyde (**3b**)

A mixture of **3g** compound 2 (0.012 mmol, 1 eq), 1.386 g phenacyl bromide (0.06 mol, 0.5 eq), and 2.48 g K_2_CO_3_ (0.018 mol, 1.5 eq) was stirred in acetone (15 cm^3^) at a room temperature for 24 h. After completion of the reaction as indicated by TLC, the reaction mixture was filtered and washed with acetone (3–15 cm^3^). The filtrate was concentrated and the residue was chromatographer on silica gel (petroleum ether:ethyl acetate 70:30, v/v) to afford the compounds (**3b**) as white solid (2.46 g, 82%). m.p.: 128–130 °C; ^1^H NMR (400 MHz, CDCl_3_): *δ* = 10.95 (s, 1H, Ar–CHO), 9.85 (s, 1H, Ar–OH), 8.05 (d, 2H, *J* = 7.93 Hz, Ar–H), 7.66–7.25 (m, 6H, Ar–H), 6.90 (d, 2H, *J* = 8.39 Hz, Ar–H), 3.97 (s, 2H, –CH_2_–) ppm; ^13^C NMR (100 MHz, DMSO-*d*_6_): *δ* = 191.2 (CHO), 182.1 (CO), 159.9 (C–OH), 154.3, 138.4, 137.5 (3C), 136.8, 134.4, 132.3, 131.1 (2C), 129.8, 128.2 (2C), 127.4, 123.5, 117.6, 112.4, 41.7 (–CH_2_–) ppm; IR (KBr): $\bar{\nu }$ = 3550, 2850, 1650, 1729 cm^−1^; MS (ESI+): *m/z* = 391.09 ([M+H]^+^), 413.0; and HRMS *m/z* calcd for C_23_H_16_ClO_4_ ([M+H]^+^) 391.07341, found 391.07316.

#### General procedure of the synthesized benzimidazole derivatives (**4a**–**u**)

*o*-Phenylenediamine, 0.068 g (0.632 mmol), was slowly added to a solution of 0.150 g (0.421 mmol) of 5-((2-benzoylbenzofuran-5-yl)methyl)-2-hydroxybenzaldehyde (**3a**) in glacial acetic acid, and the mixture was refluxed (70 °C) for 4–6 h under N_2_ atmosphere, the progress of the reaction being monitored by TLC. The mixture was cooled to room temperature, then, the mixture was poured into ice cold water and neutralised with sodium bicarbonate solution, after the mixture was washed with water and DCM for two times, the DCM layer was separated and dried over anhydrous sodium sulphate. Fatherly DCM solvent was removed in vacuo, the residue was purified by column chromatography on silica gel to give the corresponding products **4a**. The other compounds **4b**–**u** was also prepared by the similar procedure.

##### (5-(3-(1*H*-Benzo[d]imidazol-2-yl)-4-hydroxybenzyl)benzofuran-2-yl)(phenyl)methanone (**4a**)

From 0.150 g compound **3a** (0.421 mmol, 1 eq), and 0.068 g and amine (0.632 mmol, 1.5 eq), the compound **4a** was obtained as white solid (0.13 g, 87%) after purified using chromatography on a silica gel column with petroleum ether/ethyl acetate (80:20, v/v). m.p.: 236–240 °C; ^1^H NMR (400 MHz, DMSO-*d*_6_): *δ* = 13.18 (s, 1H, NH), 12.53 (s, 1H, Ar–OH), 8.0 (t, 2H, *J* = 7.28 Hz, Ar–H), 7.77–7.45 (m, 8H, Ar–H), 7.33–7.21 (m, 5H, Ar–H), 7.00 (t, 2H, *J* = 8.53 Hz), 4.05 (s, 2H, CH_2_) ppm; ^13^C NMR (100 MHz, DMSO-*d*_6_): *δ* = 182.3 (C=O), 158.3, 155.8, 153.4 (N–C–N), 151.6 (C–OH), 141.2 (2C), 138.6, 137.9, 135.3, 132.5, 131.0, 129.8 (2C) 129.5, 128.7 (2C), 123.9, 122.9 (2C), 119.0, 117.2, 118.6, 117.3, 117.2, 112.2 (2C), 41.9 (–CH_2_–) ppm; IR (KBr): $\bar{\nu }$ = 3580, 3420, 1740, 1665, 1597 cm^−1^; MS (ESI+): *m/z* = 445.08 ([M+H]^+^); and HRMS: *m/z* calcd for C_29_H_21_N_2_O_3_ ([M+H]^+^) 445.180721, found 445.180745.

##### (5-(3-(5-Bromo-1*H*-benzo[d]imidazol-2-yl)-4-hydroxybenzyl)benzofuran-2-yl)(phenyl)methanone (**4b**)

From 0.200 g compound **3a** (0.561 mmol, 1 eq), and 0.157 g amine (0.842 mmol, 1.5 eq), the compound **4b** was obtained as brown solid (0.16 g, 83%) after purified using chromatography on a silica gel column with petroleum ether/ethyl acetate (90:10, v/v). m.p.: 235–238 °C; ^1^H NMR (400 MHz, DMSO-*d*_6_): *δ* = 12.96 (s, 1H, NH), 12.49 (s, 1H, Ar–OH), 8.02 (d, 2H, *J* = 7.17 Hz, Ar–H), 7.84 (s, 1H, Ar–H), 7.62–7.29 (m, 10H, Ar–H), 7.02 (d, 1H, *J* = 8.3 Hz, Ar–H), 4.12 (s, 2H, CH_2_) ppm; ^13^C NMR (100 MHz, DMSO-*d*_6_): *δ* = 183.3 (C=O), 156.5, 154.0, 152.9 (N–C–N), 151.3 (C–OH), 140.9, 140.3, 13.0, 137.7, 135.5, 132.6, 131.9 (2C), 130.7 (2C), 128.6 (2C), 126.9, 124.7, 119.8, 118.9, 115.6, 117.9 (2C), 116.5, 112.1, 42.6 (–CH_2_–) ppm; IR (KBr): $\bar{\nu }$ = 3480, 3415, 1740, 1659, 1584 cm^−1^; MS (ESI+): *m/z* = 423.14 ([M+H]^+^); and HRMS: *m/z* calcd for C_29_H_20_BrN_2_O_3_ ([M+H]^+^) 423.13940, found 423.13905.

##### (5-(4-Hydroxy-3-(5-methyl-1*H*-benzo[d]imidazol-2-yl)benzyl)benzofuran-2-yl)(phenyl)methanone (**4c**)

From 0.180 g compound **3a** (0.505 mmol, 1 eq), and 0.093 g amine (0.758 mmol, 1.5 eq), the compound **4c** was obtained as brick red solid (0.14 g, 82%) after purified using chromatography on a silica gel column with petroleum ether/ethyl acetate (90:10, v/v). m.p.: 240–245 °C; ^1^H NMR (400 MHz, DMSO-*d*_6_): *δ* = 13.07 (s, 1H, NH), 11.76 (s, 1H, OH), 7.99 (d, 2H, *J* = 6.7 Hz, Ar–H), 7.77 (s, 1H, Ar–H), 7.75–7.68 (m, 4H, Ar–H), 7.63–7.48 (m, 5H, Ar–H), 7.43 (s, 1H, Ar–H), 7.26 (t, 1H, *J* = 8.28 Hz, Ar–H), 7.10 (d, 2H, *J* = 8.5 Hz, Ar–H), 6.99 (s, 1H, Ar–H), 4.10 (s, 2H, CH_2_), 2.45 (s, 3H, CH_3_) ppm; ^13^C NMR (100 MHz, DMSO-*d*_6_): *δ* = 183.2 (C=O), 156.9, 154.2, 153.2 (N–C–N), 151.8 (C–OH), 141.6 (2C), 138.9, 137.9, 136.7, 132.8, 131.2, 129.8, 128.4 (2C), 126.0, 122.1, 121.6, 119.4, 118.6, 117.2, 117.3, 112.5 (2C), 112.1, 42.3 (–CH_2_–) ppm; IR (KBr): $\bar{\nu }$ = 3469, 3423, 1720, 1622, 1569 cm^−1^; MS (ESI+): *m/z* = 459.1 ([M+H]^+^), and HRMS: *m/z* calcd for C_30_H_23_N_2_O_3_ ([M+H]^+^) 459.11540, found 459.11505.

##### (5-(3-(5,6-Dichloro-1*H*-benzo[d]imidazol-2-yl)-4-hydroxybenzyl)benzofuran-2-yl)(phenyl)methanone (**4d**)

From 0.200 g compound **3a** (0.561 mmol, 1 eq), and 0.148 g amine (0.842 mmol, 1.5 eq), the compound **4d** was obtained as white solid (0.15 g, 75%) after purified using chromatography on a silica gel column with petroleum ether/ethyl acetate (80:20, v/v). m.p.: 2245–250 °C; ^1^H NMR (400 MHz, DMSO-*d*_6_): *δ* = 13.20 (s, 1H, NH), 12.21 (s, 1H, OH), 8.05–7.96 (m, 4H, Ar–H), 7.78–7.68 (m, 4H, Ar–H), 7.94–7.81 (t, 2H, *J* = 7.78 Hz, Ar–H), 7.76 (s, 1H, Ar–H), 7.30 (t, 2H, *J* = 6.02 Hz, Ar–H), 7.02 (d, 1H, *J* = 8.51, Ar–H) 4.04 (s, 2H, CH_2_) ppm; ^13^C NMR (100 MHz, DMSO-*d*_6_): *δ* = 182.3 (C=O), 157.9, 156.8, 153.0, 151.8 (C–OH), 140.0, 138.7, 134.6, 131.9, 131.7, 131.0, 130.9 (2C), 129.7 (3C), 128.6 (2C), 126.9 (2C), 12.9, 121.7, 117.3, 117.0 (2C), 116.9, 116.5, 112.1, 42.3 (–CH_2_–) ppm; IR (KBr): $\bar{\nu }$ = 3415, 1720, 1569 cm^−1^; MS (ESI+): *m/z* = 445.0 ([M+H]^+^), and HRMS: *m/z* calcd for C_29_H_19_C_l2_N_2_O_3_ ([M+H]^+^) 445.09540, found 445.09515.

##### (5-(4-Hydroxy-3-(6-nitro-1*H*-benzo[d]imidazol-2-yl)benzyl)benzofuran-2-yl)(phenyl)methanone (**4e**)

From 0.200 g compound **3a** (0.561 mmol, 1 eq), and 0.129 g amine (0.842 mmol, 1.5 eq), the compound **4e** was obtained as yellow solid (0.16 g, 83%) after purified using chromatography on a silica gel column with petroleum ether/ethyl acetate (90:10, v/v). m.p.: 230–235 °C; ^1^H NMR (400 MHz, DMSO-*d*_6_): *δ* = 13.74 (s, 1H, NH), 11.81 (s, 1H, OH), 8.3 (d, 2H, *J* = 8.1 Hz, Ar–H), 7.99–7.76 (m, 4H, Ar–H), 7.72–7.69 (m, 5H, Ar–H), 7.65 (d, 3H, Ar–H) ppm; ^13^C NMR (100 MHz, DMSO-*d*_6_): *δ* = 182.3 (C=O), 156.9, 155.8, 152.9 (N–C–N), 150.2 (C–OH), 147.9, 143.7 (C–NO_2_), 139.8, 138.2, 137.9, 134.6, 131.9, 130.0, 129.7 (3C), 128.5 (2C), 123.9, 119.8, 118.9, 118.4, 116.9, 116.2, 113.0, 112.0, 42.0 (–CH_2_–) ppm; IR (KBr): $\bar{\nu }$ = 3510, 3450, 1785, 1665, 1587 cm^−1^; MS (ESI+): *m/z* = 490.0 ([M+H]^+^), and HRMS: *m/z* calcd for C_29_H_20_N_3_O_5_ ([M+H]^+^) 490.10145, found 490.10175.

##### (5-(3-(5-Fluoro-1*H*-benzo[d]imidazol-2-yl)-4-hydroxybenzyl)benzofuran-2-yl)(phenyl)methanone (**4f**)

From 0.180 g compound **3a** (0.505 mmol, 1 eq), and 0.092 g amine (0.758 mmol, 1.5 eq), the compound **4f** was obtained as off white solid (0.15 g, 85%) after purified using chromatography on a silica gel column with petroleum ether/ethyl acetate (90:10, v/v). m.p.: 235–238 °C; ^1^H NMR (400 MHz, DMSO-*d*_6_): *δ* = 13.21 (s, 1H, NH), 12.52 (s, 1H, OH), 8.02 (d, 3H, *J* = 7.2 Hz, Ar–H), 7.80–7.41 (m, 8H, Ar–H), 7.42–6.89 (m, 4H, Ar–H), 4.01 (s, 2H, CH_2_) ppm; ^13^C NMR (100 MHz, DMSO-*d*_6_): *δ* = 182.1 (C=O), 159.1, 156.7, 154.1, 153.1, 151.4 (C–OH), 144.2, 137.9, 137.5, 136.7, 135.5, 132.4, 132.0, 131.9, 131.0, 129.8 (2C), 128.7 (2C), 128.3, 125.4 (2C), 119.7, 118.5, 117.4, 117.3, 117.1, 112.1, 109.9, 102.3, 42.1 (–CH_2_–) ppm; IR (KBr): $\bar{\nu }$ = 3453, 3405, 2550, 1735, 1655, 1587 cm^−1^; MS (ESI+): *m/z* = 463.1 ([M+H]^+^); and HRMS: *m/z* calcd for C_29_H_20_FN_2_O_3_ ([M+H]^+^) 463.14446, found 463.14142.

##### (5-(3-(5,6-Dimethyl-1*H*-benzo[d]imidazol-2-yl)-4-hydroxybenzyl)benzofuran-2-yl)(phenyl)methanone (**4g**)

From 0.250 g compound **3a** (0.702 mmol, 1 eq), and 0.145 g amine (1.053 mmol, 1.5 eq), the compound **4g** was obtained as brick red solid (0.187 g, 75%) after purified using chromatography on a silica gel column with petroleum ether/ethyl acetate (90:10, v/v). m.p.: 250–255 °C; ^1^H NMR (400 MHz, DMSO-*d*_6_): *δ* = 12.95 (s, 1H, NH), 9.81 (s, 1H, OH), 8.01–7.90 (m, 4H, Ar–H), 7.77–7.65 (m, 7H, Ar–H), 7.53–7.17 (m, 3H, Ar–H), 6.95 (s, 1H, Ar–H), 4.10 (s, 2H, CH_2_), 2.31 (s, 6H, CH_3_) ppm; ^13^C NMR (100 MHz, DMSO-*d*_6_): *δ* = 183.0 (C=O), 156.4, 154.0, 152.6 (N–C–N), 151.5 (C–OH), 142.8 (2C), 138.9, 137.7, 136.7, 132.1, 131.9, 129.1, 128.6 (2C), 126.1, 122.9, 122.5, 119.4, 118.6, 117.2, 117.0, 112.5 (2C), 112.1, 45.2, 21.0 (2C) ppm; IR (KBr): $\bar{\nu }$ = 3480, 3413, 2550, 1724, 1645, 1567 cm^−1^; MS (ESI+): *m/z* = 473.2 ([M+H]^+^); and HRMS: *m/z* calcd for C_31_H_25_N_2_O_3_ ([M+H]^+^) 473.18430, found 473.18597.

##### (5-(3-(5-Chloro-1*H*-benzo[d]imidazol-2-yl)-4-hydroxybenzyl)benzofuran-2-yl)(phenyl)methanone (**4h**)

From 0.200 g compound **3a** (0.561 mmol, 1 eq), and 0.199 g amine (0.842 mmol, 1.5 eq), the compound **4h** was obtained as off white color solid (154 g, 78%) after purified using chromatography on a silica gel column with petroleum ether/ethyl acetate (90:10, v/v). m.p.: 235–240 °C; ^1^H NMR (400 MHz, DMSO-*d*_6_): *δ* = 13.12 (s, 1H, NH), 11.59 (s, 1H, OH), 8.02 (d, 2H, *J* = 7.5 Hz, Ar–H), 7.66–7.15, (m, 11H, Ar–H), 6.91 (d, 2H, *J* = 7.25 Hz, Ar–H), 3.94 (s, 2H, –CH_2_–) ppm; ^13^C NMR (100 MHz, DMSO-*d*_6_): *δ* = 183.0 (C=O), 156.5, 155.1, 152.9, 150.1 (C–OH), 140.2, 138.9, 137.7, 132.9, 132.6, 131.9, 131.0, 129.2, 129.7 (2C), 127.6 (2C), 124.9, 119.2, 118.1, 115.3 (2C), 112.1, 107.9, 101.8, 42.3 ppm; IR (KBr): $\bar{\nu }$ = 3458, 3430, 2570, 1775, 1655, 1597 cm^−1^; MS (ESI+): *m/z* = 509.21 ([M+H]^+^); and HRMS: *m/z* calcd for C_29_H_20_ClN_2_O_3_ ([M+H]^+^) 509.19560, found 509.19515.

##### (5-(3-(1*H*-Benzo[d]imidazol-2-yl)-4-hydroxybenzyl)benzofuran-2-yl)(4-chlorophenyl)methanone (**4i**)

From 0.200 g compound **3b** (0.512 mmol, 1 eq), and 0.083 g amine (0.769 mmol, 1.5 eq), the compound **4i** was obtained as white solid (0.168 g, 84%) after purified using chromatography on a silica gel column with petroleum ether/ethyl acetate (90:10, v/v). m.p.: 240–242 °C; ^1^H NMR (400 MHz, DMSO-*d*_6_): *δ* = 13.32 (s, 1H, NH), 12.02 (s, 1H, OH), 8.0 (t, 2H, *J* = 7.28 Hz, Ar–H), 7.79 (s, 1H, Ar–H), 7.77–7.45 (m, 8H, Ar–H), 7.33–7.21 (s, 1H, Ar–H), 7.00 (t, 2H, *J* = 8.53 Hz, Ar–H), 4.05 (s, 2H, CH_2_) ppm; ^13^C NMR (100 MHz, DMSO-*d*_6_): *δ* = 183.9 (C=O), 56.8, 154.5, 152.1, 151.9 (C–OH), 141.7 (2C), 138.0, 137.2, 135.7, 133.4, 132.6, 131.4 (3C), 129.6, 129.1 (2C), 127.5, 126.6 (2C), 123.4, 117.7, 117.5 (2C), 113.0 (2C), 112.6, 43.8 (–CH_2_–) ppm; IR (KBr): $\bar{\nu }$ = 3580, 3423, 2570, 1740, 1565, 1597 cm^−1^; MS (ESI+): *m/z* = 478.09 ([M+H]^+^); and HRMS: *m/z* calcd for C_29_H_20_ClN_2_O_3_ ([M+H]^+^) 478.10960, found 478.10944.

##### (5-(3-(6-Bromo-1*H*-benzo[d]imidazol-2-yl)-4-hydroxybenzyl)benzofuran-2-yl)(4-chlorophenyl)methanone (**4j**)

From 0.150 g compound **3b** (0.384 mmol, 1 eq), and 0.106 g amine (0.576 mmol, 1.5 eq), the compound **4j** was obtained as white solid (0.124 g, 83%) after purified using chromatography on a silica gel column with petroleum ether/ethyl acetate (70:30, v/v). m.p.: 235–240 °C; ^1^H NMR (400 MHz, DMSO-*d*_6_): *δ* = 13.00 (s, 1H, NH), 12.75 (s, 1H, OH), 8.02 (d, 2H, *J* = 7.17 Hz, Ar–H), 7.66 (s, 1H, Ar–H), 7.62–7.35 (m, 9H, Ar–H), 6.92 (d, 1H, *J* = 8.8 Hz, Ar–H), 4.13 (s, 2H, CH_2_) ppm; ^13^C NMR (100 MHz, DMSO-*d*_6_): *δ* = 182.2 (C=O), 156.7, 155.1, 153.9 (N–C–N), 150.8 (C–OH), 139.9 (2C), 138.0, 137.9 (2C), 134.6, 132.7, 131.0 (3C), 129.7 (2C), 126.0, 121.2, 119.9, 116.9 (2C), 116.8, 112.5, 109.6, 102.9, 42.3 (–CH_2_–) ppm; IR (KBr): $\bar{\nu }$ = 3580, 3425, 2570, 1740, 1560, 1585 cm^−1^; MS (ESI+): *m/z* = 557.0 ([M+H]^+^); and HRMS: *m/z* calcd for C_29_H_19_BrClN_2_O_3_ ([M+H]^+^) 557.01960, found 557.01944.

##### (4-Chlorophenyl)(5-(4-hydroxy-3-(6-methyl-1*H*-benzo[d]imidazol-2-yl)benzyl)benzofuran-2-yl)methanone (**4k**)

From 0.180 g compound **3b** (0.461 mmol, 1 eq), and 0.084 g amine (0.692 mmol, 1.5 eq), the compound **4k** was obtained as brown solid (0.153 g, 85%) after purified using chromatography on a silica gel column with petroleum ether/ethyl acetate (80:20, v/v). m.p.: 250–255 °C; ^1^H NMR (400 MHz, DMSO-*d*_6_): *δ* = 13.04 (s, 1H, NH), 11.55 (s, 1H, OH), 8.01 (d, 2H, *J* = 6.7 Hz, Ar–H), 7.79 (s, 1H, Ar–H), 7.74–7.49 (m, 7H, Ar–H), 7.38 (s, 1H, Ar–H), 7.26 (d, 1H, *J* = 8.03 Hz, Ar–H), 7.09 (t, 1H, *J* = 4.08 Hz, Ar–H), 6.97 (s, 1H, Ar–H), 4.10 (s, 2H, CH_2_), 2.45 (s, 3H, CH_3_) ppm; ^13^C NMR (100 MHz, DMSO-*d*_6_): *δ* = 183.9 (C=O), 156.8, 152.1, 152.0, 151.6 (C–OH), 138.0, 137.2, 136.4, 135.8, 133.4, 132.6, 132.4, 131.4 (3C), 130.2 (2C), 129.6, 126.7, 123.4, 117.7, 117.5, 116.9, 113.0, 112.6, 43.6 (–CH_2_–), 21.5 (–CH_3_) ppm; IR (KBr): $\bar{\nu }$ = 3395, 2700, 1720, 1622, 1570 cm^−1^; MS (ESI+): *m/z* = 493.10 ([M+H]^+^); and HRMS: *m/z* calcd for C_30_H_23_ClN_2_O_3_ ([M+H]^+^) 493.10960, found 493.10904.

##### (4-Chlorophenyl)(5-(3-(5,6-dichloro-1*H*-benzo[d]imidazol-2-yl)-4-hydroxybenzyl) benzo furan-2-yl)methanone (**4l**)

From 0.200 g compound **3b** (0.512 mmol, 1 eq), and 0.134 g amine (0.769 mmol, 1.5 eq), the compound 41 was obtained as brick red solid (0.17 g, 85%) after purified using chromatography on a silica gel column with petroleum ether/ethyl acetate (70:30, v/v). m.p.: 250–255 °C; ^1^H NMR (400 MHz, DMSO-*d*_6_): *δ* = 13.20 (s, 1H, NH), 12.00 (s, 1H, OH), 8.05 (d, 3H, *J* = 8.2 Hz, Ar–H), 7.79 (s, 1H, Ar–H), 7.74–7.63 (m, 4H, Ar–H), 7.50 (d, 1H, *J* = 8.2, Ar–H), 7.30 (d, 1H, *J* = 8.2, Hz, Ar–H), 7.01 (d, 1H, *J* = 8.2 Hz, Ar–H), 4.10 (s, 2H, CH_2_) ppm; ^13^C NMR (100 MHz, DMSO-*d*_6_): *δ* = 182.2 (C=O), 156.8, 155.2, 153.1, 150.0 (C–OH), 139.5, 136.4, 135.9, 130.9, 131.1 (3C), 130.2 (2C), 129.5 (C), 129.0 (2C), 128.6 (2C), 124.2 (C), 119.5 (C), 118.0, 117.3 (2C), 116.0, 112.0 (C), 43.0 (–CH_2_–) ppm; IR (KBr): $\bar{\nu }$ = 3423, 2500, 1720, 1622, 1595 cm^−1^; MS (ESI+): *m/z* = 547.10 ([M+H]^+^); and HRMS: *m/z* calcd for C_30_H_18_ClN_2_O_3_ ([M+H]^+^) 547.11960, found 547.11904.

##### (4-Chlorophenyl)(5-(4-hydroxy-3-(6-nitro-1*H*-benzo[d]imidazol-2-yl)benzyl)benzofuran-2-yl)methanone (**4m**)

From 0.150 g compound **3b** (0.384 mmol, 1 eq), and 0.088 g amine (0.576 mmol, 1.5 eq), the compound **4m** was obtained as pale yellow (0.127 g, 85%) after purified using chromatography on a silica gel column with petroleum ether/ethyl acetate (90:10, v/v). m.p.: 250–255 °C; ^1^H NMR (400 MHz, DMSO-*d*_6_): *δ* = 13.74 (s, 1H, NH), 11.90 (s, 1H, OH), 8.3 (d, 2H, *J* = 8.1 Hz, Ar–H), 8.00–7.76 (m, 4H, Ar–H), 7.68–7.69 (m, 4H, Ar–H), 7.65 (d, 3H, Ar–H), 4.12, (s, 2H, CH_2_) ppm; ^13^C NMR (100 MHz, DMSO-*d*_6_): *δ* = 182.2 (C=O), 156.4, 155.0, 153.8 (N–C–N), 150.9 (C–OH), 149.2, 140.3 (C–NO_2_), 139.8, 138.2 (C–Cl), 137.6, 136.2, 132.9, 131.2 (3C), 130.0, 129.2 (2C), 126.0, 119.0, 118.8, 116.9 (2C), 116.1, 112.5, 112.0, 42.2 (–CH_2_–) ppm; IR (KBr): $\bar{\nu }$ = 3510, 3450, 2590, 1790, 1664, 1600 cm^−1^; MS (ESI+): *m/z* = 524.10 ([M+H]^+^); and HRMS: *m/z* calcd for C_29_H_19_ClN_3_O_5_ ([M+H]^+^) 524.10660, found 524.10604.

##### (4-Chlorophenyl)(5-(3-(6-fluoro-1*H*-benzo[d]imidazol-2-yl)-4-hydroxybenzyl)benzofuran-2-yl)methanone (**4n**)

From 0.150 g compound **3b** (0.384 mmol, 1 eq), and 0.072 g amine (0.576 mmol, 1.5 eq), the compound **4n** was obtained as white solid (0.120 g, 80%) after purified using chromatography on a silica gel column with petroleum ether/ethyl acetate (80:20, v/v). m.p.: 240–254 °C; ^1^H NMR (400 MHz, DMSO-*d*_6_): *δ* = 13.02 (s, 1H, NH), 12.52 (s, 1H, Ar–OH, D_2_O exchangeable), 8.55 (d, 3H, *J* = 7.2 Hz, Ar–H), 7.80–7.46 (m, 7H, Ar–H), 7.45–6.85 (m, 4H, Ar–H), 4.00 (s, 2H, CH_2_) ppm; ^13^C NMR (100 MHz, DMSO-*d*_6_): *δ* = 183.2 (C=O), 156.9, 155.1, 150.9 (N–C–N), 150.3 (C–OH), 139.9, 138.0, 137.5 (2C), 134.6, 132.0, 131.0 (3C), 129.4 (2C), 126.0, 121.4, 119.9, 116.5 (2C), 116.8, 112.0, 109.5, 102.0, 42.0 (–CH_2_–) ppm; IR (KBr): $\bar{\nu }$ = 3460, 3423, 1735, 1655, 1587 cm^−1^; MS (ESI+): *m/z* = 463.21 ([M+H]^+^); and HRMS: *m/z* calcd for C_29_H_19_ClFN_2_O_3_ ([M+H]^+^) 463.19608, found 463.19642.

##### (4-Chlorophenyl)(5-(3-(5,6-dimethyl-1*H*-benzo[d]imidazol-2-yl)-4-hydroxybenzyl) benzo furan-2-yl)methanone (**4o**)

From 0.200 g compound **3b** (0.512 mmol, 1 eq), and 0.104 g amine (0.769 mmol, 1.5 eq) the compound **4o** was obtained as brick red (0.15 g, 75%) after purified using chromatography on a silica gel column with petroleum ether/ethyl acetate (70:30, v/v). m.p.: 250–255 °C; ^1^H NMR (400 MHz, DMSO-*d*_6_): *δ* = 12.95 (s, 1H, NH), 10.81 (s, 1H, OH), 8.01–7.90 (m, 4H, Ar–H), 7.77–7.70 (m, 6H, Ar–H), 7.53–6.98 (m, 3H, Ar–H), 6.95 (s, 1H, Ar–H), 4.09 (s, 2H, CH_2_), 2.38 (s, 6H, 2CH_3_) ppm; ^13^C NMR (100 MHz, DMSO-*d*_6_): *δ* = 182.5 (C=O), 157.0, 156.8, 153.0 (N–C–N), 151.6 (C–OH), 137.9 (C–Cl), 137.6 (2C), 136.0, 134.5, 132.8 (2C), 132.0, 131.1 (2C), 128.5 (2C), 127.0, 124.8, 120.0, 118.7, 116.3 (2C), 113.9 (2C), 112.6, 42.2 (–CH_2_–), 21.0 (2C) ppm; IR (KBr): $\bar{\nu }$ = 3480, 3423, 1724, 1645, 1567 cm^−1^; MS (ESI+): *m/z* = 507.8 ([M+H]^+^); and HRMS: *m/z* calcd for C_31_H_24_ClN_2_O_3_ ([M+H]^+^) 507.86082, found 507.86020.

##### (5-(3-(6-Chloro-1*H*-benzo[d]imidazol-2-yl)-4-hydroxybenzyl)benzofuran-2-yl)(4-chlorophenyl)methanone (**4p**)

From 0.150 g compound **3b** (0.384 mmol, 1 eq), and 0.081 g amine (0.576 mmol, 1.5 eq), the compound **4p** was obtained as light red solid (0.112 g, 75%) after purified using chromatography on a silica gel column with petroleum ether/ethyl acetate (90:10, v/v). m.p.: 235–237 °C; ^1^H NMR (400 MHz, DMSO-*d*_6_): *δ* = 13.12 (s, 1H, NH), 12.00 (s, 1H, OH), 8.02 (d, 2H, *J* = 7.5 Hz, Ar–H), 7.66–7.00 (m, 10H, Ar–H), 7.00 (d, 2H, *J* = 7.25 Hz, Ar–H), 4.00 (s, 2H, CH_2_) ppm; ^13^C NMR (100 MHz, DMSO-*d*_6_): *δ* = 182.2 (C=O), 155.9, 154.1, 153.4, 151.4, 137.9, 137.6 (2C), 135.3, 132.8, 132.1 (3C), 131.0, 129.9 (2C), 128.8 (2C), 126.9, 122.9, 118.3, 117.2 (3C), 116.6, 112.6, 42.0 (–CH_2_–) ppm; IR (KBr): $\bar{\nu }$ = 3458, 3400, 2570, 1795, 1655, 1597 cm^−1^; MS (ESI+): *m/z* = 513.1 ([M+H]^+^); and HRMS: *m/z* calcd for C_29_H_19_Cl_2_N_2_O_3_ ([M+H]^+^) 513.16082, found 513.16020.

##### (5-(4-Hydroxy-3-(1*H*-naphtho[2,3-d]imidazol-2-yl)benzyl)benzofuran-2-yl)(phenyl)methanone (**4q**)

From 0.200 g compound **3a** (0.561 mmol, 1 eq), and 0.133 g amine (0.842 mmol, 1.5 eq) the compound **4q** was obtained as white solid (0.172 g, 86%) after purified using chromatography on a silica gel column with petroleum ether/ethyl acetate (90:10, v/v). m.p.: 255–260 °C; ^1^H NMR (400 MHz, DMSO-*d*_6_): *δ* = 13.21 (s, 1H, NH), 9.97 (s, 1H, OH), 8.23 (s, 1H, Ar–H), 8.11 (s, 2H, Ar–H), 8.08–7.96 (m, 2H, Ar–H), 7.79–7.58 (m, 5H, Ar–H), 7.52 (t, 3H, *J* = 6.78 Hz, Ar–H), 7.51 (t, 1H, *J* = 5.7 Hz, Ar–H), 7.42 (s, 1H, Ar–H), 7.35 (s, 1H, Ar–H), 7.04 (d, 1H, *J* = 8.2 Hz, Ar–H), 4.18 (s, 2H, CH_2_) ppm; ^13^C NMR (100 MHz, DMSO-*d*_6_): *δ* = 183.1 (C=O), 156.3, 154.0, 152.9 (N–C–N), 151.5 (C–OH), 141.7 (2C), 138.2, 137.1, 136.7, 132.7, 131.9, 129.1, 128.6 (2C), 127.5 (2C), 127.0 (2C), 126.1, 122.9 (2C), 119.4, 118.6, 117.2, 117.0, 112.9 (2C), 112.2, 42.1 (–CH_2_–) ppm; IR (KBr): $\bar{\nu }$ = 3420, 2450, 1724, 1645,1547 cm^−1^; MS (ESI+): *m/z* = 495.23 ([M+H]^+^); and HRMS: *m/z* calcd for C_33_H_23_N_2_O_3_ ([M+H]^+^) 495.17081, found 495.17032.

##### (5-(4-Hydroxy-3-(1*H*-imidazo[4,5-b]pyridin-2-yl)benzyl)benzofuran-2-yl)(phenyl)methanone (**4r**)

From 0.150 g compound **3a** (0.421 mmol, 1 eq), and 0.068 g amine (0.632 mmol, 1.5 eq), the compound **4r** was obtained as white solid (0.120 g, 80%) after purified using chromatography on a silica gel column with petroleum ether/ethyl acetate (90:10, v/v). m.p.: 240–245 °C; ^1^H NMR (400 MHz, DMSO-*d*_6_): *δ* = 13.84 (s, 1H, NH), 12.83 (s, 1H, OH), 8.18–7.95 (m, 4H, Ar–H), 7.81–7.62 (m, 4H, Ar–H), 7.65-7.57 (t, 2H, Ar–H), 7.52 (d, 1H, *J* = 8.7 Hz, Ar–H), 7.32 (s, 2H, Ar–H), 7.01 (t, 1H, *J* = 8.2 Hz, Ar–H), 4.09 (s, 2H, CH_2_) ppm; ^13^C NMR (100 MHz, DMSO-*d*_6_): *δ* = 182.3 (C=O), 157.9, 153.2 (C–OH), 154.3, 151.4, 150.5, 144.5, 137.8, 136.9, 136.7, 135.3, 131.0, 130.3, 129.7, 129.7 (2C), 127.9 (2C), 126.9, 123.1, 121.8, 118.4, 117.7, 116.9 (2C), 112.2 (2C), 44.3 (–CH_2_–) ppm; IR (KBr): $\bar{\nu }$ = 3450, 3420, 1725, 1645, 1557 cm^−1^; MS (ESI+): *m/z* = 446.19 ([M+H]^+^); and HRMS: *m/z* calcd for C_28_H_20_N_3_O_3_ ([M+H]^+^) 446.11531, found 446.11570.

##### (5-(3-(5-Bromo-1H-imidazo[4,5-b]pyridin-2yl)4hydroxybenzyl)benzofuran-2-yl)(phenyl)methanone (**4s**)

From 0.180 g compound **3a** (0.505 mmol, 1 eq), and 0.141 g amine (0.758 mmol, 1.5 eq), the compound **4s** was obtained as yellow solid (0.140 g, 78%) after purified using chromatography on a silica gel column with petroleum ether/ethyl acetate (90:10, v/v). m.p.: 230–235 °C; ^1^H NMR (400 MHz, DMSO-*d*_6_): *δ* = 13.22 (s, 1H, NH), 12.58 (s, 1H, OH), 8.05 (s, 3H, Ar–H), 7.80–7.38 (m, 8H, Ar–H), 7.28 (s, 1H, Ar–H), 7.12 (d, 1H, Ar–H), 7.01 (s, 1H, Ar–H), 4.04 (s, 2H, CH_2_) ppm; ^13^C NMR (100 MHz, DMSO-*d*_6_): *δ* = 183.3 (C=O), 156.1, 154.0, 153.4, 151.6, 137.4, 136.7, 134.5, 134.1, 132.9, 132.2, 131.9, 130.0, 129.8 (2C), 129.0 (2C), 128.6 (2C), 126.4, 122.9, 121.7, 117.3, 116.9, 112.1, 42.3 (–CH_2_–) ppm; IR (KBr): $\bar{\nu }$ = 3580, 3423, 2650, 1730, 1651, 1554 cm^−1^; MS (ESI+): *m/z* = 423.99 ([M+H]^+^); and HRMS: *m/z* calcd for C_28_H_19_BrN_3_O_3_ ([M+H]^+^) 423.72551, found 423.72571.

##### (4-Chlorophenyl)(5-(4-hydroxy-3-(3*H*-imidazo[4,5-b]pyridin-2-yl)benzyl)benzofuran-2-yl)methanone (**4t**)

From 0.200 g compound **3b** (0.512 mmol, 1 eq), and 0.083 g amine (0.769 mmol, 1.5 eq), the compound **4t** was obtained as white solid (0.160 g, 80%) after purified using chromatography on a silica gel column with petroleum ether/ethyl acetate (90:10, v/v). m.p.: 240–245 °C; ^1^H NMR (400 MHz, DMSO-*d*_6_): *δ* = 13.85 (s, 1H, NH), 12.80 (s, 1H, Ar–OH, D_2_O exchangeable), 8.40 (m, 1H, Ar–H), 8.20–8.00 (m, 4H, Ar–H), 7.85–7.67 (m, 3H, Ar–H), 7.40–7.20 (d, 3H, *J* = 8.6 Hz, Ar–H), 7.38 (m, 2H, Ar–H), 4.05 (s, 2H, CH_2_) ppm; ^13^C NMR (100 MHz, DMSO-*d*_6_): δ = 182.2 (C=O), 159.2, 154.1, 153.1 (C–OH), 151.4, 150.5, 144.3 (C=N), 137.9, 137.6, 136.7, 135.3, 131.0, 130.9, 129.9, 129.5 (2C), 128.3 (2C), 126.9, 122.9, 122.0, 118.5, 117.3, 117.2 (2C), 112.2, 44.0 (–CH_2_–) ppm; IR (KBr): $\bar{\nu }$ = 3450, 3425,1725, 1645, 1557 cm^−1^; MS (ESI+): *m/z* = 480.1 ([M+H]^+^); and HRMS: *m/z* calcd for C_28_H_19_ClN_3_O_3_ ([M+H]^+^) 480.09541, found 480.09581.

##### (5-(3-(5-Bromo-3*H*-imidazo[4,5-b]pyridin-2-yl)-4-hydroxybenzyl)benzofuran-2-yl)(4-chloro phenyl)methanone (**4u**)

From 0.250 g compound **3b** (0.641 mmol, 1 eq), and 0.178 g amine (0.961 mmol, 1.5 eq), the compound **4u** was obtained as light brown solid (0.195 g, 78%) after purified using chromatography on a silica gel column with petroleum ether/ethyl acetate (90:10, v/v). m.p.: 230–235 °C; ^1^H NMR (400 MHz, DMSO-*d*_6_): *δ* = 13.29 (s, 1H, NH), 12.35 (s, 1H, OH), 8.03 (s, 4H, Ar–H), 7.83–7.63 (m, 5H, Ar–H), 7.43 (t, 2H, Ar–H), 7.33 (s, 1H, Ar–H), 7.02 (d, 1H, *J* = 6.5, Ar–H), 4.04 (s, 2H, CH_2_) ppm; ^13^C NMR (100 MHz, DMSO-*d*_6_): *δ* = 182.1 (C=O), 159.1, 155.4, 153.1, 151.6, 142.5, 140.1, 137.9, 134.5, 133.9, 132.9, 132.6, 131.9 (2C), 129.1 (2C), 129.8 (2C), 128.7 (2C), 126.9, 122.9, 119.4, 117.4, 117.3, 117.0, 112.9, 42.6 (–CH–) ppm; IR (KBr): $\bar{\nu }$ = 3580, 3435, 2650, 1730, 1651, 1554 cm^−1^; MS (ESI+): *m/z* = 557.0 ([M+H]^+^); and HRMS: *m/z* calcd for C_28_H_18_BrClN_3_O_3_ ([M+H]^+^) 557.02551, found 557.02621.

### In vitro antimicrobial assay

Antimicrobial activity was evaluated using agar well diffusion method. The activity was determined by measuring the diameter of the inhibition zone (in mm). Samples of the tested compounds (50 μL, 1 mg/mL concentration) were loaded into the wells on the plates. All solutions were prepared in DMSO and pure DMSO was loaded as control. The plates were kept for incubation at 35 °C for 1–5 days and then were examined for the formation of inhibition zone. Each inhibition zone was measured three times to get an average value. The test was performed three times for each bacterium culture [26–28].

### Minimal inhibitory concentration (MIC) measurement

The microorganism’s susceptibility tests in nutrient and potato dextrose broths were used for the determination of MIC. Stock 1000 μg/mL solutions of the tested compounds, ciprofloxacin and nystatin were prepared in DMSO followed by dilutions to 250–25 μg/mL concentrations. Inoculated microorganism suspensions were incubated at 37 °C for 1–5 days for MIC determination. The microorganism suspensions were inoculated into the concentrations of corresponding compounds and control experiments and listed in Tables 1 and 2 respectively.

### Evaluation of cell cytotoxicity

Hela, Supt1 cancer cell lines were used to evaluate the impact on cell viability of each compound. Hela cells were maintained in DMEM, Supt1 cells in RPMI, all the mediums were supplemented with 10% fetal bovine serum (FBS) cells were maintained in keratinocyte serum free media with 0.1 ng/mL human recombinant EGF, 0.05 mg/mL bovine pituitary extract and additional CaCl_2_ 44.1 mg/mL (final concentration 0.4 mM), 2 mM l-glutamine at 37 °C under a 5% CO_2_ atmosphere. For each cell line, 70% confluent cell culture flask was trypsinized and were seeded cells in a 96-well plate at a density of 5000 cells by well in the appropriate complete media; These cells were treated with increasing concentrations of drugs, and incubated for 24 h. The cells were washed with media and resuspended in new medium. To this, 20 mL of 5 mg/mL MTT (Sigma-Aldrich) was added and incubated for **4f**. The medium was removed from cells, and dissolved in DMSO (DMSO 0.1% in phosphate saline buffer) and read in an ELISA micro plate reader at 570 nm; 48 h after treatment, viability was accessed by MTT assay.

## Conclusions

In conclusion, the present work offers the promotion of a simple procedure in an inexpensive route for the synthesis of benzimidazole derivatives (**4a**–**u**) via condensation of 5-((2-benzoylbenzofuran-5-yl)methyl)-2hydroxybenzaldehyde with ortho phenylenediamine under conventional heating. The biological evolution exhibited that these molecules (**4a**–**u**) were good and selective against bacterial and fungal strains in the micro molar range. The experimental antimicrobial studies resulted that the compounds (**4f**) and (**4u**) are good inhibitors for antibacterial and antifungal activities respectively. The compounds of (**4f**) and (**4u**) have proven potential against cancer cell lines. Interestingly, the compound concentration increases nature of cytotoxicity also increasing. Molecular docking study revealed that not only hydrogen bonding interactions, π–π stacking, π–cation and Mn metal co-ordination interactions were also favourable for its antibacterial activity.

## Additional file

**Additional file 1.** Predicted ADME (Drug-likeness) properties of all synthesized compounds and selected copies of spectrum (^1^H/^13^C-NMR, MS and HRMS) for synthesized some benzimidazole derivatives are provided in supporting information.
