# Supplementary material for: Synthesis, anti-microbial activity, cytotoxicity of some novel substituted (5-(3-(1H-benzo[d]imidazol-2-yl)-4-hydroxybenzyl)benzofuran-2-yl)(phenyl)methanone analogs
Source: Chem Cent J. 2018 Jan 9;12:1. doi: 10.1186/s13065-017-0364-3 (PMC5760494; doi:10.1186/s13065-017-0364-3)
Supplement: Supplementary file 1 — Additional file 1. Predicted ADME (Drug-likeness) properties of all synthesized compounds and selected copies of spectrum (1H/13C-NMR, MS and HRMS) for synthesized some benzimidazole derivatives are provided in supporting information. [file 13065_2017_364_MOESM1_ESM.docx]

**Synthesis, anti-microbial activity, cytotoxicity of some novel substituted (5-(3-(1H-benzo[d]imidazol-2-yl)-4-hydroxy benzyl) benzofuran-2-yl)(phenyl)methanone analogs**

Bhookya Shankar ^1^, Jalapathi Pochampally ^1, *^, Balabadra Saikrishna ^2^, Shaym perugu ^3^, Vijjulatha Manga ^2^

**^1^**Department of chemistry, University College of Science, Osmania University, Saifabad, Hyderabad- 500004, India

**^2^**Molecular Modeling and Medicinal Chemistry Group, Department of Chemistry, Osmania University, Hyderabad, Telangana-500 007, India

**^3^**Biomedical Informatics Center, National Institute of Nutrition, Hyderabad-500 007, Telangana, India

Pochampally Jalapathi

[pochampalli.ou.chemi@gmail.com](mailto:pochampalli.ou.chemi@gmail.com)

1) Predicted ADME properties: Page number **2-3**

2) Specters’ **4-13**

***Supporting Information***

***Predicted ADME properties:***

QikProp module of Schrodinger was used computationally to evaluate (absorption, distribution, metabolism and excretion) properties of synthesized compounds. The physically significant descriptors and pharmaceutically relevant properties of synthesized molecules with compliance to Lipinski’s rule of five are calculated by QikProp 3.0.

Drug-likeness of newly synthesized compounds was analyzed by applying Lipinski's rule of five (**Table 4** provided in supplementary material). The Lipinski’s rule for the drug-like molecules, states that the molecule should have molecular weight <650 Da, H-bond donors <5, H-bond acceptors <10, and a log P of <5. For the synthesized compounds, the partition coefficient (QPlogPo/w) and water solubility (QPlogS) is critical for estimating the absorption and distribution of drugs within the body, which ranged between 5.06–7.19 and 7.32–9.98, respectively. Crossing the blood-brain barrier (BBB), which is a prerequisite for the entry of drugs into CNS, was originated to be in the acceptable range (0.72–1.34) (except 4e, 4m). Caco-2 cell permeability (QPPCaco), a model governing gut-blood barrier, ranged from 398.96 to 821.53 (except 4e, 4m). MDCK cell permeability (QPPMDCK), a model that mimics the blood-brain barrier, ranges from 199.8 to 4664 (except 4e, 4m). Further, the predicted percentage human oral absorption, ranges from 82.11 to 100 (except 4e, 4m). All these pharmacokinetic parameters were found to be good and acceptable range.

**Table 4**

ADME properties of synthesized compounds

| Mol | M.Wt | QPlogPo/w^a^ | QPlogS^b^ | QPP  Caco^c^ | QPlog  BB^d^ | QPP  MDCK^e^ | %Human Oral Absorption^f^ |
| --- | --- | --- | --- | --- | --- | --- | --- |
| 4a | 444.5 | 5.77 | -7.74 | 805.16 | -1.02 | 391.4 | 100 |
| 4b | 523.4 | 6.34 | -8.73 | 761.88 | -0.91 | 978.4 | 89.74 |
| 4c | 458.5 | 6.08 | -8.45 | 761.26 | -1.1 | 368.4 | 100 |
| 4d | 513.4 | 6.69 | -9.23 | 761.55 | -0.8 | 1887 | 91.8 |
| 4e | 489.5 | 5.08 | -7.93 | 96.756 | -2.24 | 39.63 | 79.27 |
| 4f | 462.5 | 5.88 | -7.98 | 705.6 | -0.98 | 611.8 | 100 |
| 4g | 472.5 | 6.35 | -8.87 | 761.54 | -1.12 | 368.5 | 100 |
| 4h | 478.9 | 6.24 | -8.49 | 749.45 | -0.91 | 893.9 | 100 |
| 4i | 478.9 | 6.26 | -8.47 | 806.03 | -0.87 | 967.5 | 100 |
| 4j | 557.8 | 6.84 | -9.48 | 761.14 | -0.76 | 2413 | 92.64 |
| 4k | 493 | 6.58 | -9.2 | 761.11 | -0.95 | 909.3 | 100 |
| 4l | 547.8 | 7.19 | -9.98 | 762.25 | -0.64 | 4664 | 94.72 |
| 4m | 523.9 | 5.58 | -8.69 | 96.617 | -2.11 | 97.69 | 69.21 |
| 4n | 496.9 | 6.49 | -8.85 | 804.66 | -0.77 | 1741 | 100 |
| 4o | 507 | 6.85 | -9.62 | 768.66 | -0.96 | 919.1 | 92.77 |
| 4p | 513.4 | 6.75 | -9.22 | 804.96 | -0.72 | 2375 | 92.57 |
| 4q | 494.5 | 6.71 | -8.95 | 821.53 | -1.07 | 400 | 100 |
| 4r | 445.5 | 5.06 | -7.32 | 432.14 | -1.34 | 199.8 | 90.76 |
| 4s | 524.4 | 5.67 | -8.2 | 473.37 | -1.14 | 605.1 | 82.11 |
| 4t | 479.9 | 5.54 | -8.04 | 432.95 | -1.19 | 494.2 | 93.62 |
| 4u | 558.8 | 6 | -8.78 | 398.96 | -1.07 | 1232 | 82.73 |

* Predicted **^a^**partition coefficient,**^b^**water solubility, **^c^**Caco-2 cell permeability, **^d^**Crossing the blood-brain barrier, **^e^**MDCK cell permeability, **^f^**percentage human oral absorption

**^1^H NMR spectrum of 5-((2-benzoylbenzofuran-5-yl)methyl)-2-hydroxybenzaldehyde (3a):**

**^13^C NMR spectrum of 5-((2-benzoylbenzofuran-5-yl)methyl)-2-hydroxybenzaldehyde (3a):**

**
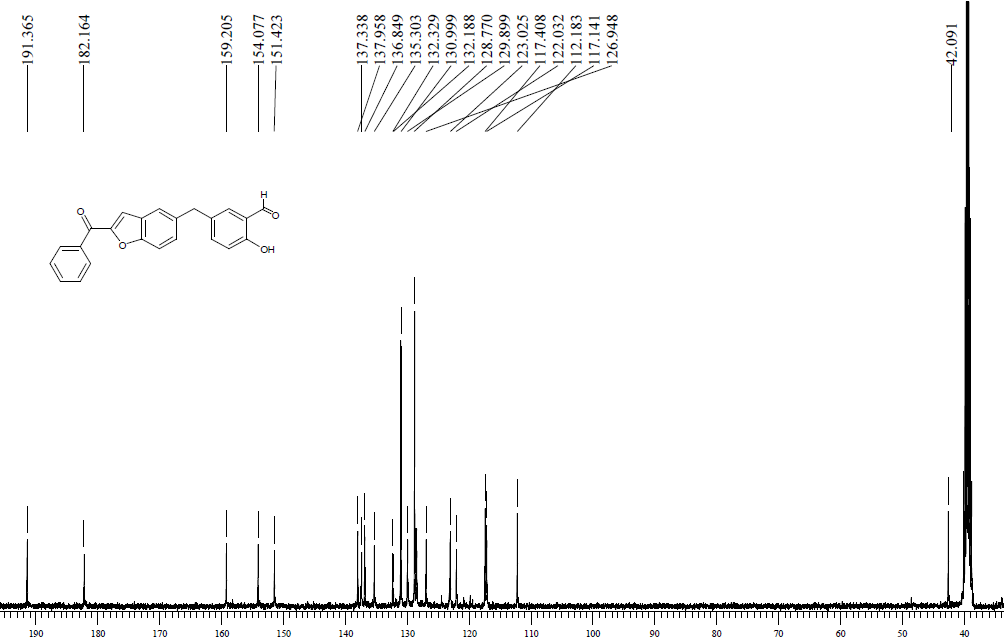
**

**HRMS spectrum of 5-((2-benzoylbenzofuran-5-yl)methyl)-2-hydroxybenzaldehyde (3a):**

**
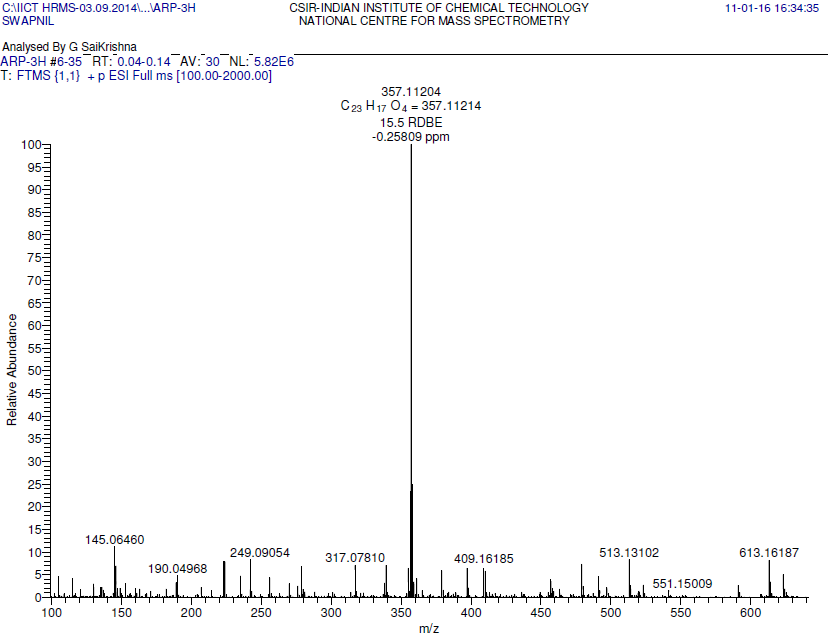
**

**^1^H NMR spectrum of 5-((2-(4-chlorobenzoyl)benzofuran-5-yl)methyl)-2-hydroxybenzalde- hyde (3b):**


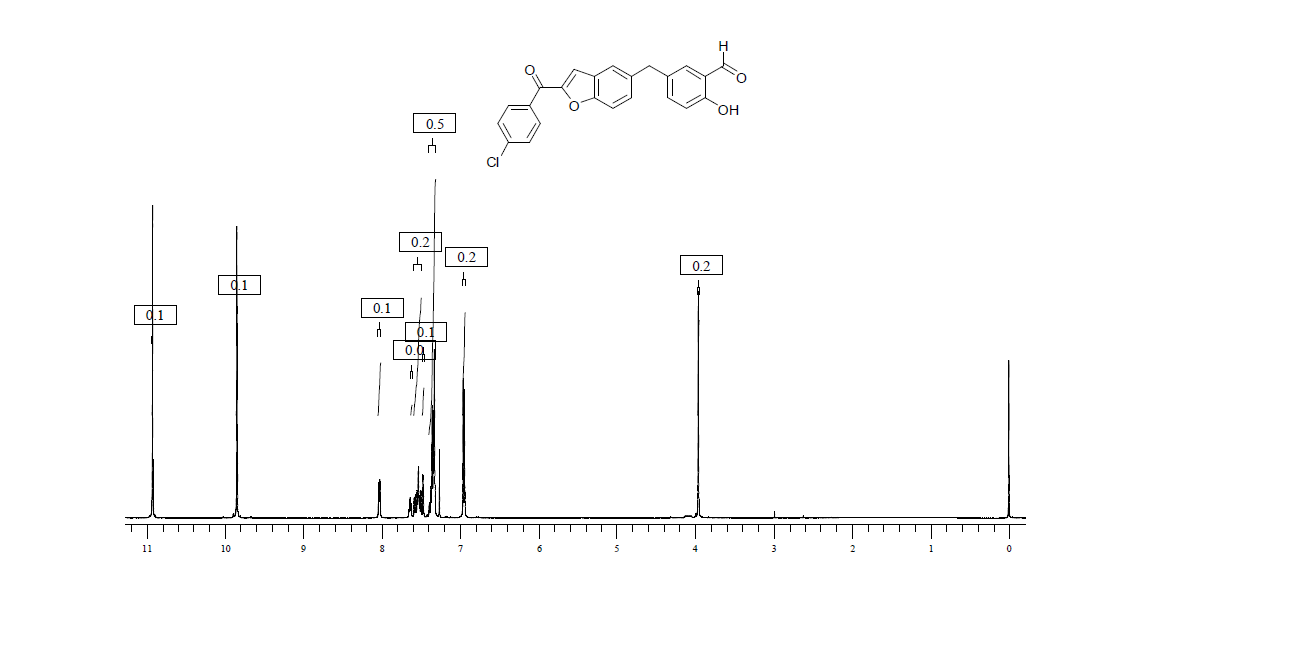

**^13^C-NMR spectrum of 5-((2-(4-chlorobenzoyl) benzofuran-5-yl) methyl)-2-hydroxybenza- ldehyde (3b):**
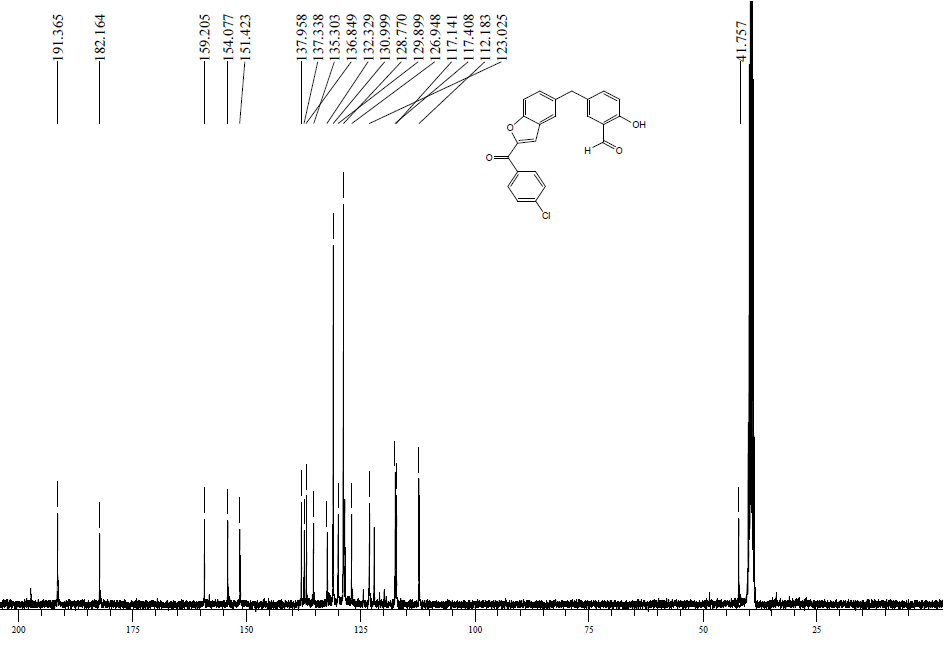

**HRMS spectrum of 5-((2-(4-chlorobenzoyl) benzofuran-5-yl) methyl)-2-hydroxybenzaldehy - de (3b):**


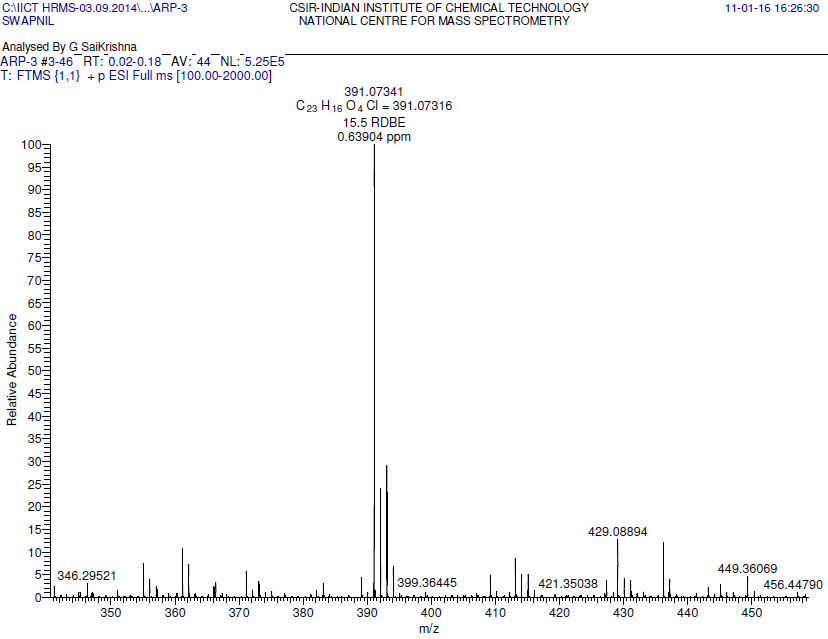

**^1^H NMR spectrum of (5-(3-(1*H*-benzo[d]imidazol-2-yl)-4-hydroxybenzyl)benzofuran-2-yl) (phenyl)methanone (4a):**

**
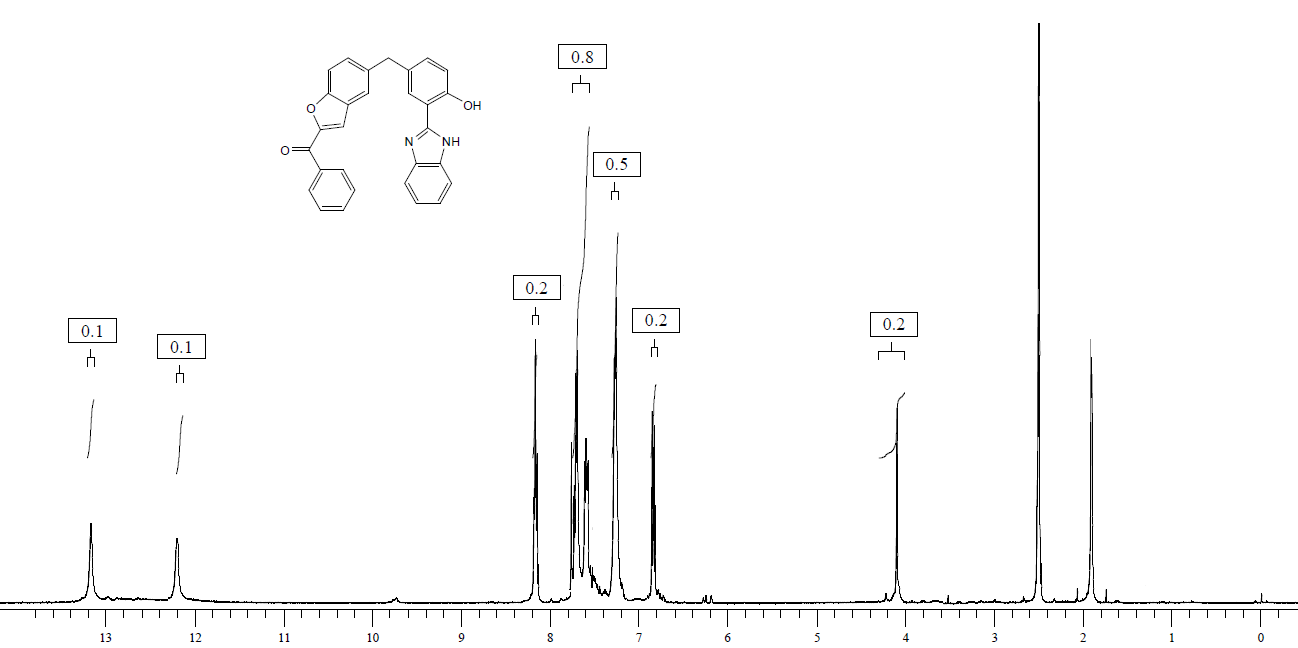
**

**^13^C NMR spectrum of (5-(3-(1*H*-benzo[d]imidazol-2-yl)-4-ydroxybenzyl)benzofuran-2-yl) (phenyl)methanone (4a):**

**
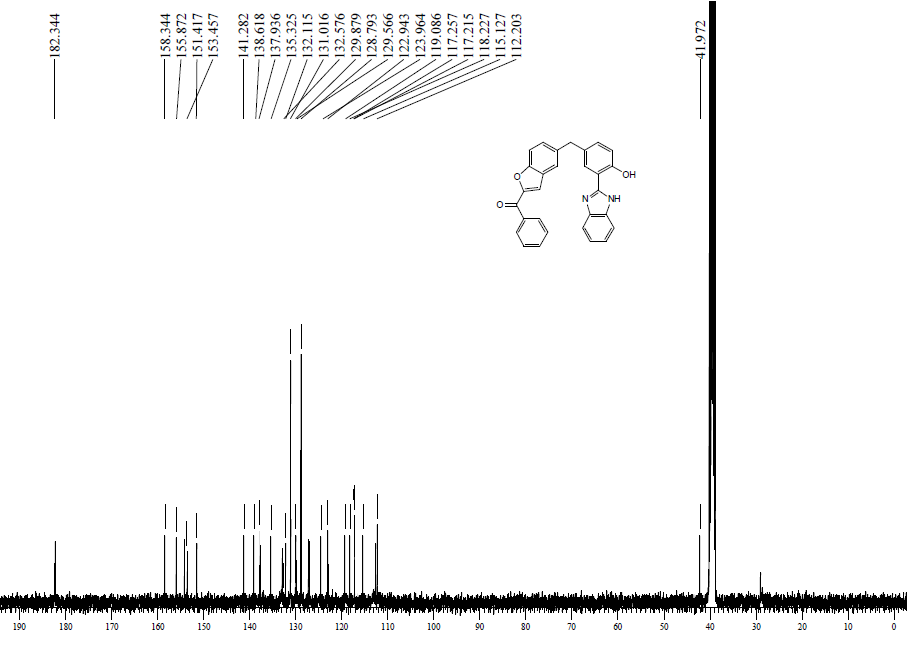
**

**Mass spectrum of (5-(3-(1*H*-benzo[d]imidazol-2-yl)-4-hydroxybenzyl)benzofuran-2-yl) (phenyl)methanone (4a):**

**^1^H NMR spectrum of (5-(3-(5-fluoro-1*H*-benzo[*d*]imidazol-2-yl)-4-hydroxybenzyl)benzo furan-2-yl)(phenyl)methanone(4f):**


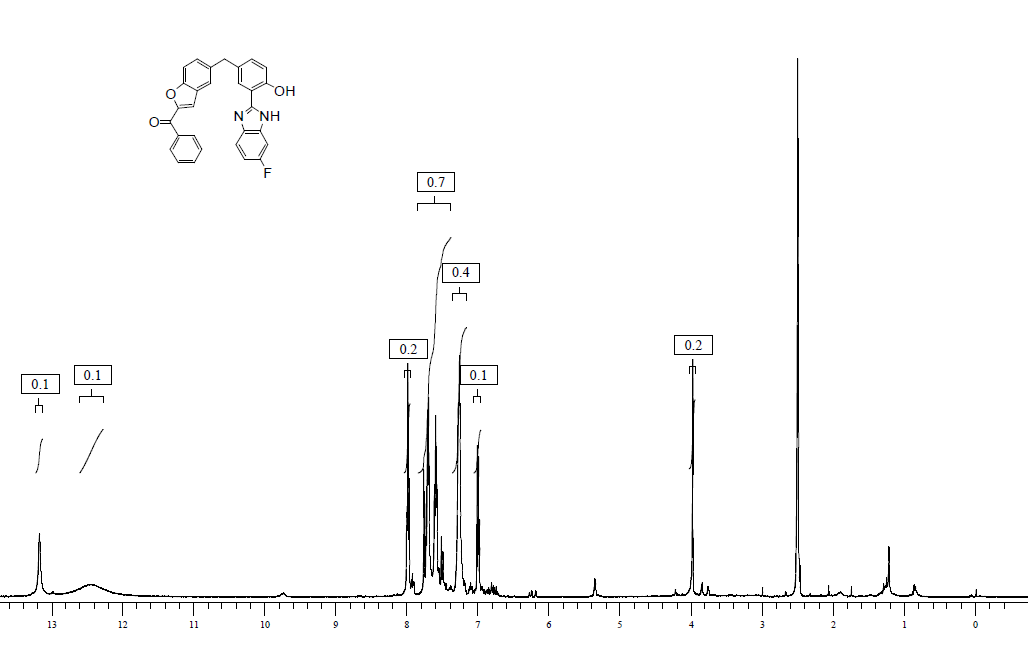

**^13^C NMR spectrum of (5-(3-(5-fluoro-1*H*-benzo[*d*]imidazol-2-yl)-4-hydroxybenzyl)benzo furan-2-yl)(phenyl)methanone(4f):**


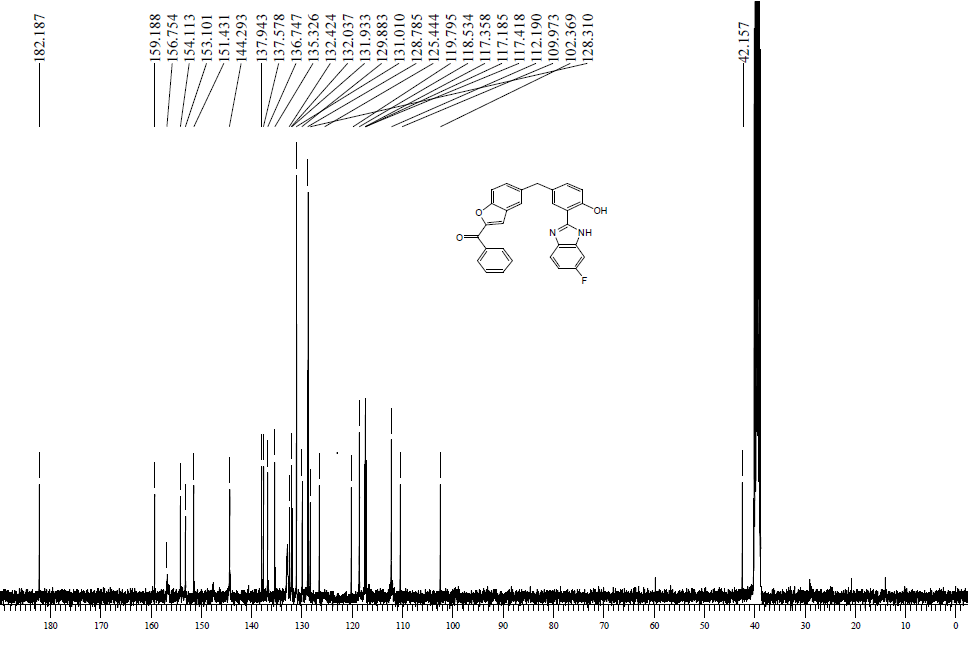

**HRMS spectrum of (5-(3-(5-fluoro-1*H*-benzo[*d*]imidazol-2-yl)-4-hydroxybenzyl)benzo furan-2-yl) (phenyl)methanone(49f):**

**
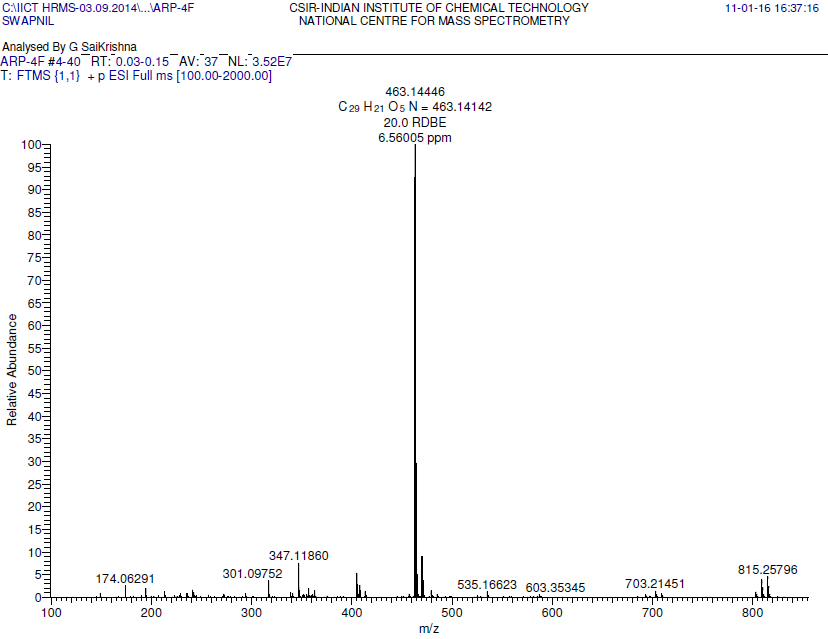
**

**^1^H NMR Spectrum of (5-(3-(6-bromo-1*H*-benzo[*d*]imidazol-2-yl)-4-hydroxybenzyl) benzofuran-2-yl)(4-chlorophenyl)methanone (4u):**

**
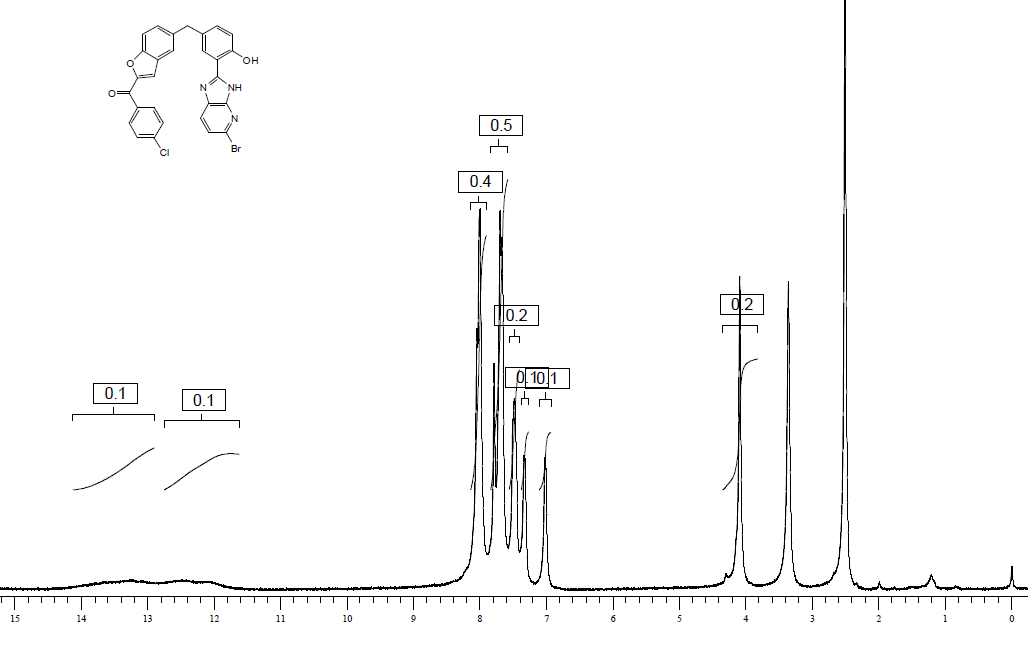
**

**^13^C NMR Spectrum of (5-(3-(6-bromo-1*H*-benzo[*d*]imidazol-2-yl)-4-hydroxybenzyl) benzofuran-2-yl)(4-chlorophenyl)methanone (4u):**

**
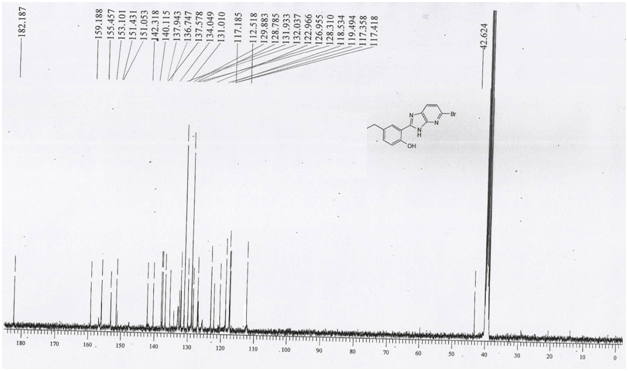
**

**HRMS Spectrum of (5-(3-(6-bromo-1*H*-benzo[*d*]imidazol-2-yl)-4-hydroxybenzyl) benzo furan-2-yl) (4-chlorophenyl)methanone (4u):**

**
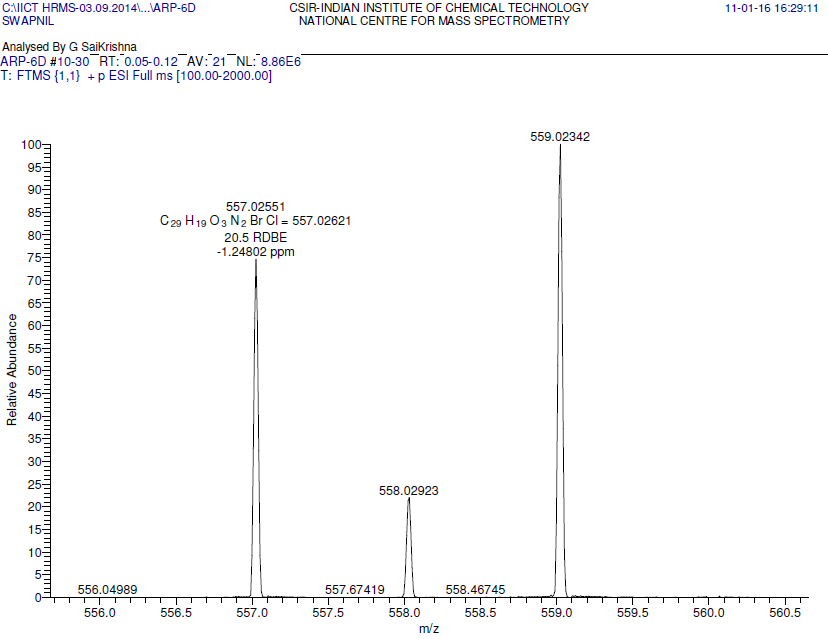
**

**^1^H NMR Spectrum of (4-chlorophenyl)(5-(4-hydroxy-3-(3*H*-imidazo[4,5-*b*]pyridin-2-yl) benzyl)benzofuran-2-yl)methanone (4t):**

**
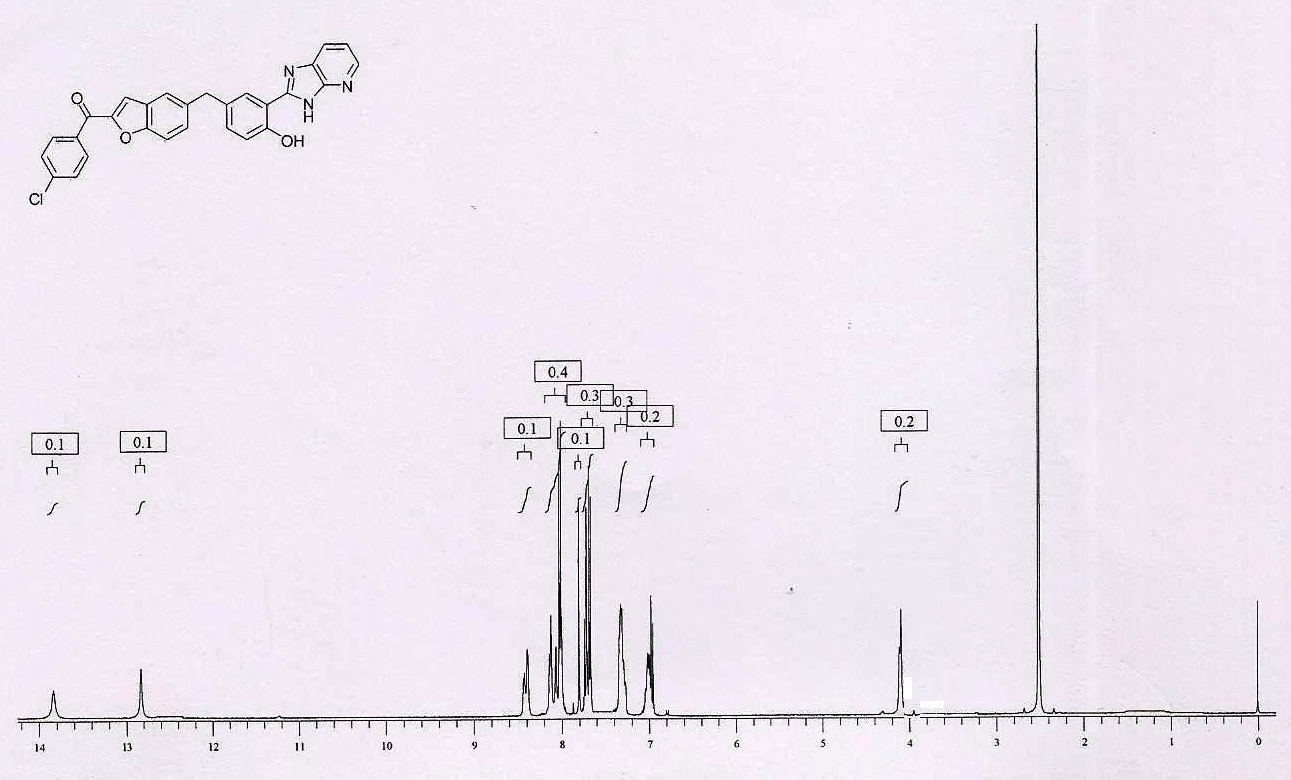
**

**^13^C NMR Spectrum of (4-chlorophenyl)(5-(4-hydroxy-3-(3*H*-imidazo[4,5-*b*]pyridin-2-yl) benzyl)benzofuran-2-yl)methanone (4t):**

**
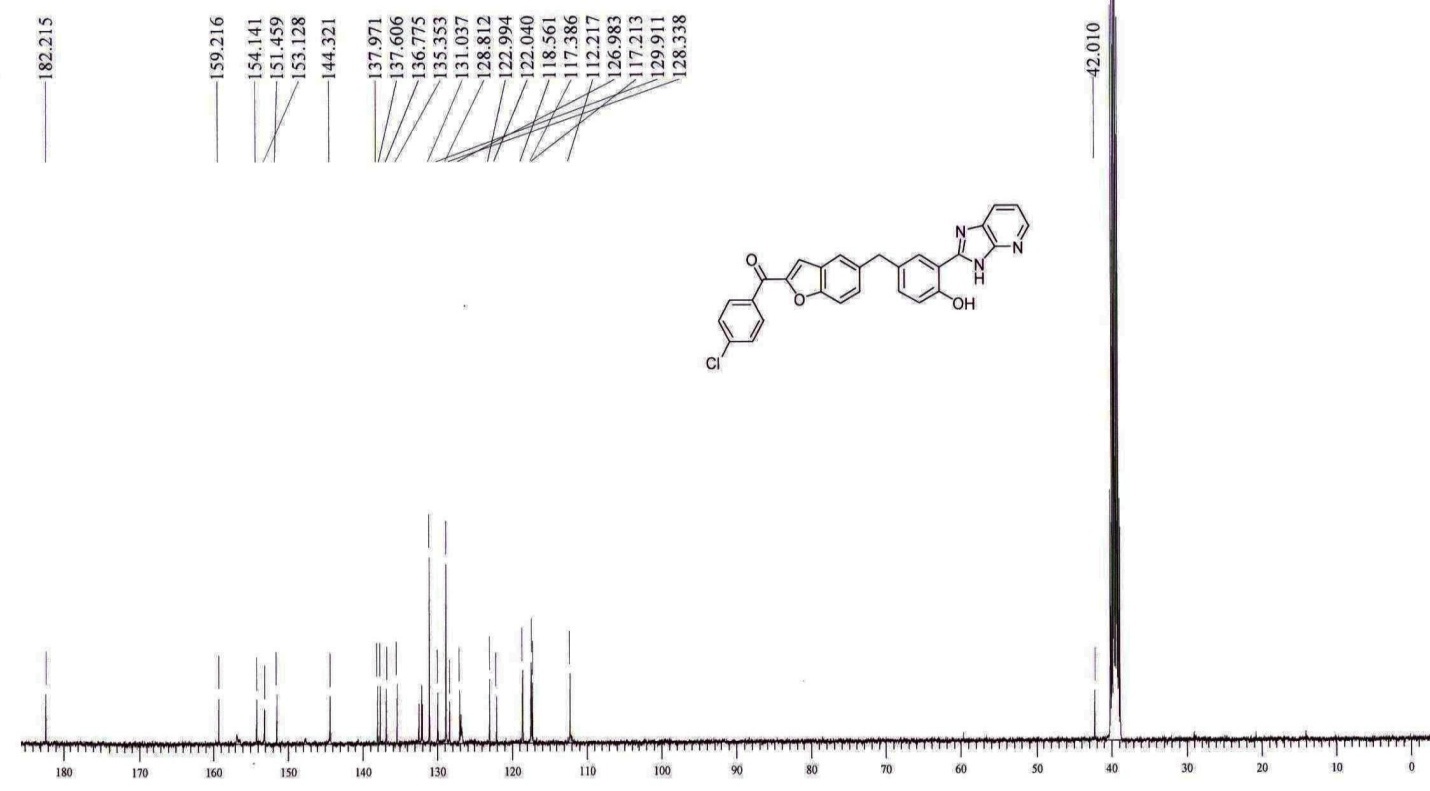
**

**Mass spectrum of (4-chlorophenyl)(5-(4-hydroxy-3-(3*H*-imidazo[4,5-*b*] pyridin-2-yl) benzyl) benzofuran-2-yl)methanone (4t):**

**
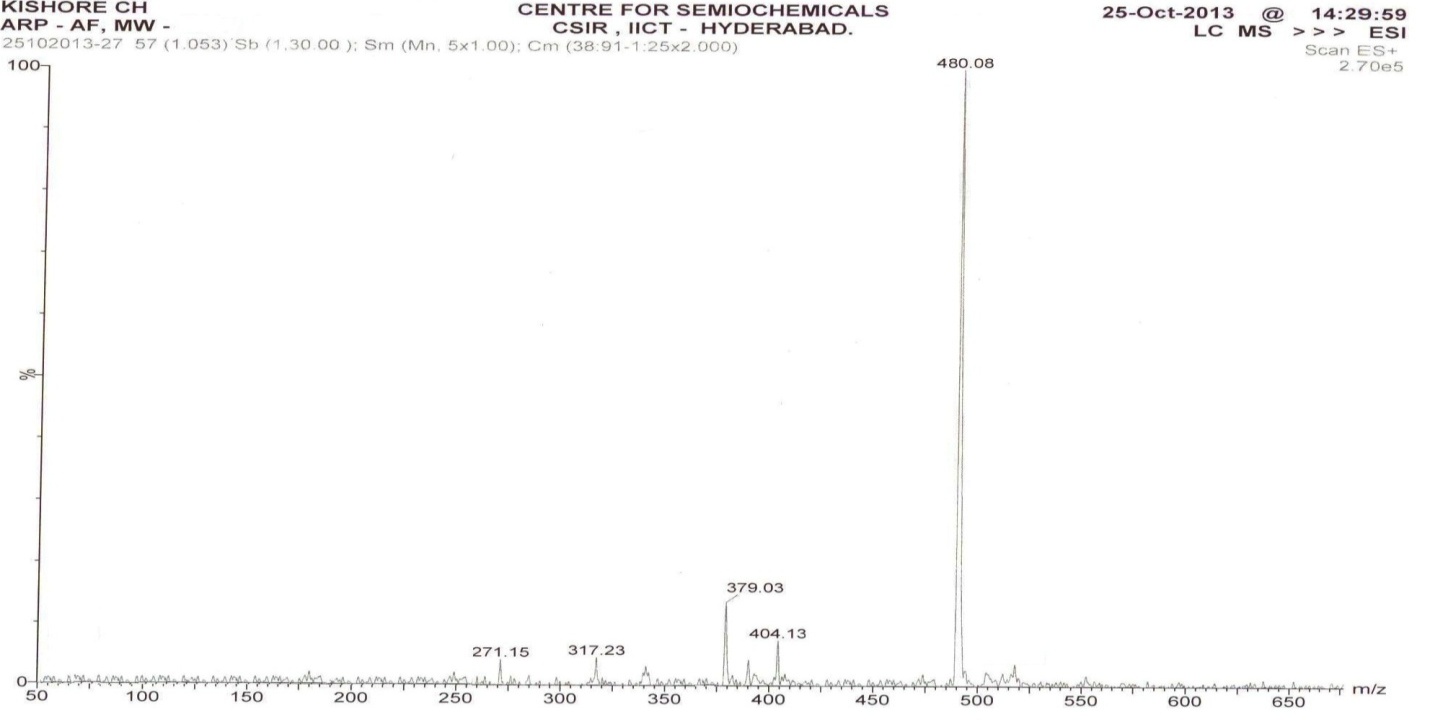
**

**HRMS spectrum of (5-(3-(5,6-dimethyl-1*H*-benzo[*d*]imidazol-2-yl)4hydroxybenzyl) benzofuran-2-yl) (phenyl)methanone(4g):**
